# Supplementary figures and images for: An atypical NLR protein modulates the NRC immune receptor network in Nicotiana benthamiana
Source: PLoS Genet. 2023 Jan 19;19(1):e1010500. doi: 10.1371/journal.pgen.1010500 (PMC9851556; doi:10.1371/journal.pgen.1010500)

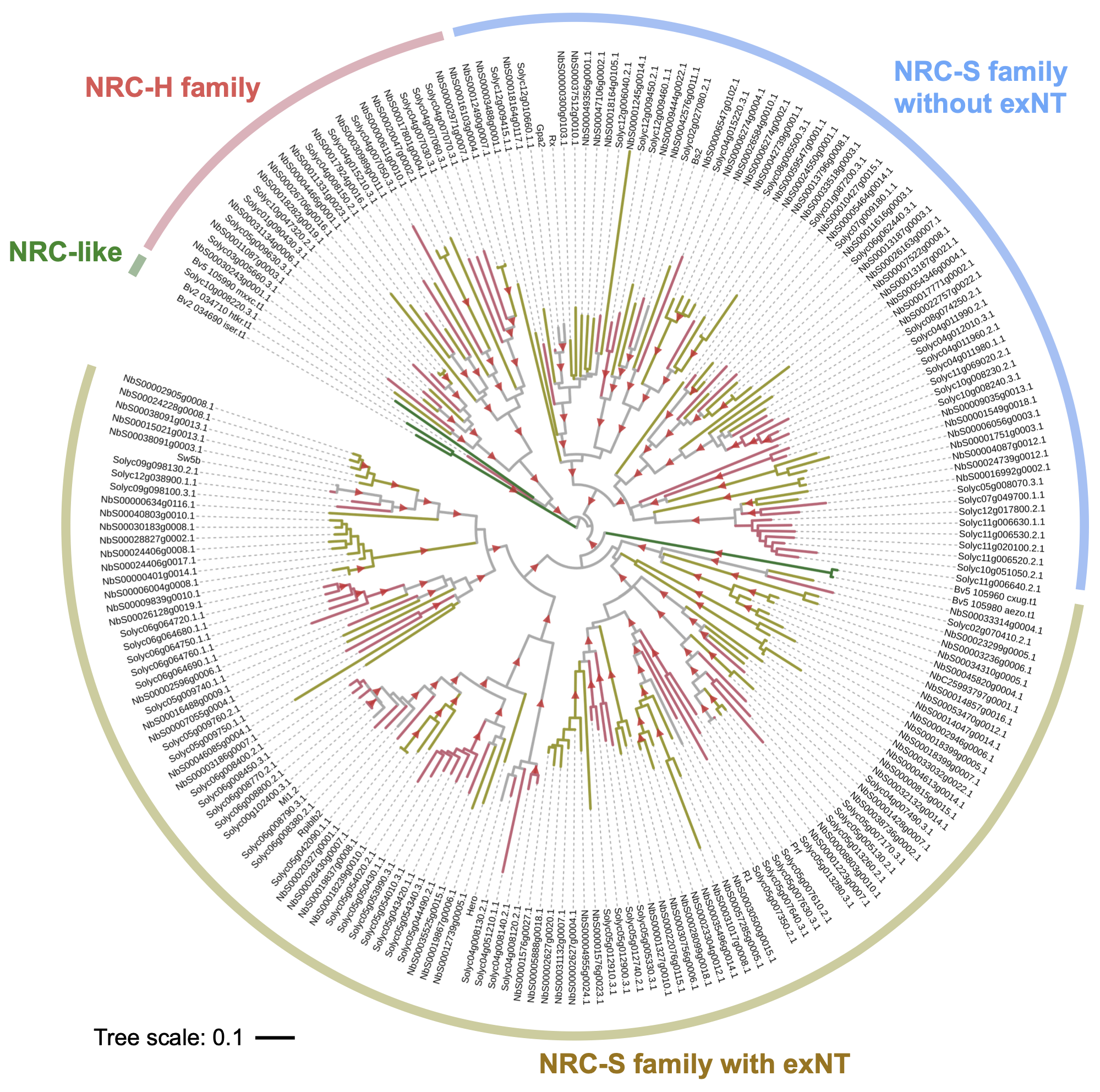

Supplement: S1 Fig — NRC-sensor (NRC-S) and NRC-helper (NRC-H) proteins identified in Adachi et al. [24] were used for the MAFFT multiple alignment and phylogenetic analyses. The phylogenetic tree was constructed with the NB-ARC domain sequences in MEGA7 by the neighbour-joining method. Each leaf is labelled with different colour ranges indicating plant species, N. benthamiana (NbS-), tomato (Solyc-) and sugar beet (Bv-). The NRC-S clade is divided into NLRs that lack an extended N-terminal domain (exNT) prior to their CC domain and those that carry an exNT. Red arrow heads indicate bootstrap support > 0.7. The scale bars indicate the evolutionary distance in amino acid substitution per site. (TIF) [file pgen.1010500.s001.tif]

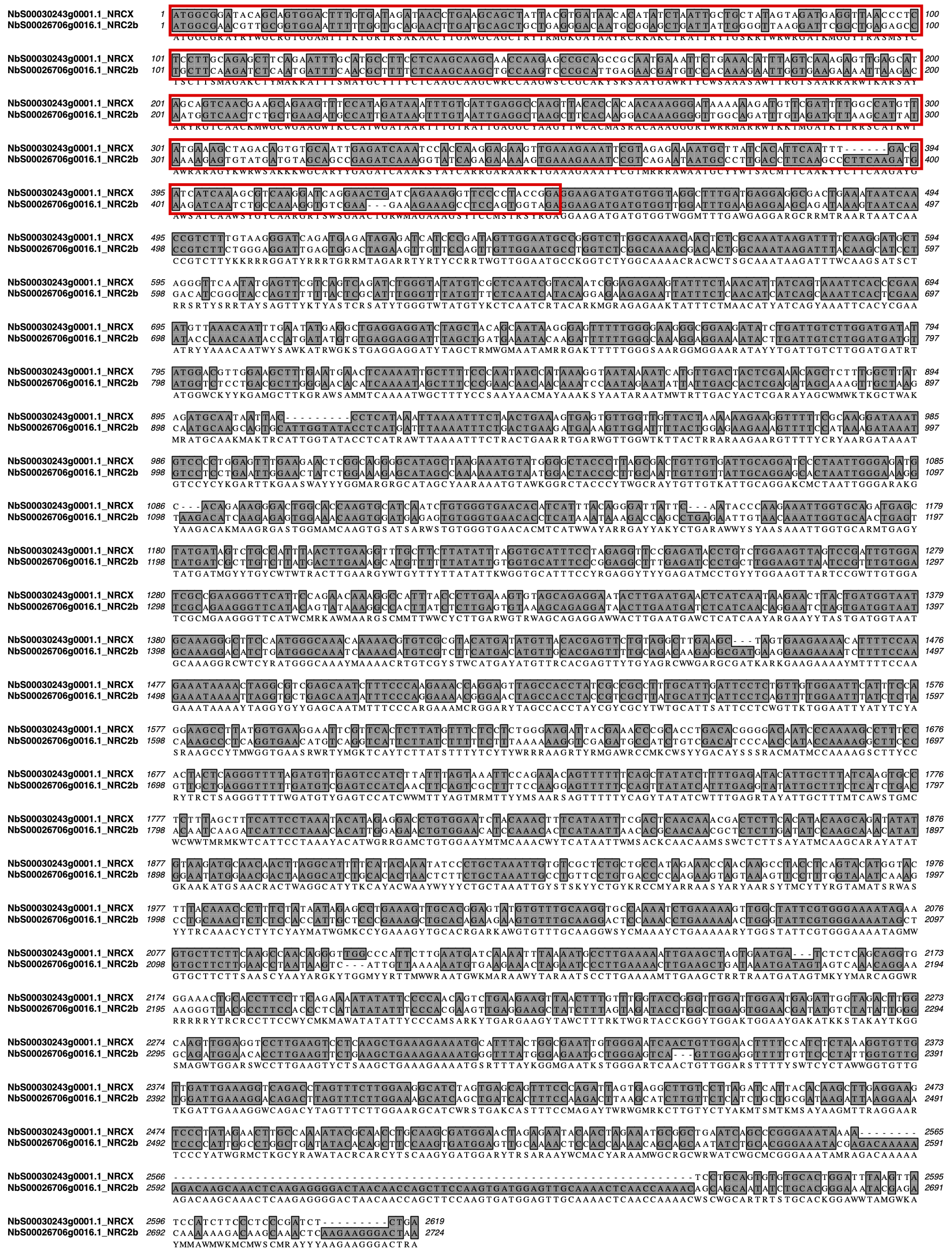

Supplement: S2 Fig — cDNA sequences of NRCX (NbS00030243g0001.1) and a closely related paralog gene NRC2b (NbS00026706g0016.1) were used for the MAFFT multiple alignment. Red boxes indicate the region used for making virus-induced gene silencing and hairpin RNA constructs of NRCX. Search of the NRCX 446-bp sequence in SGN VIGS Tool (https://vigs.solgenomics.net/) does not hit off-target candidate genes in Nicotiana benthamiana v0.4.4 and Nicotiana benthamiana v1.0.1 databases. (TIF) [file pgen.1010500.s002.tif]

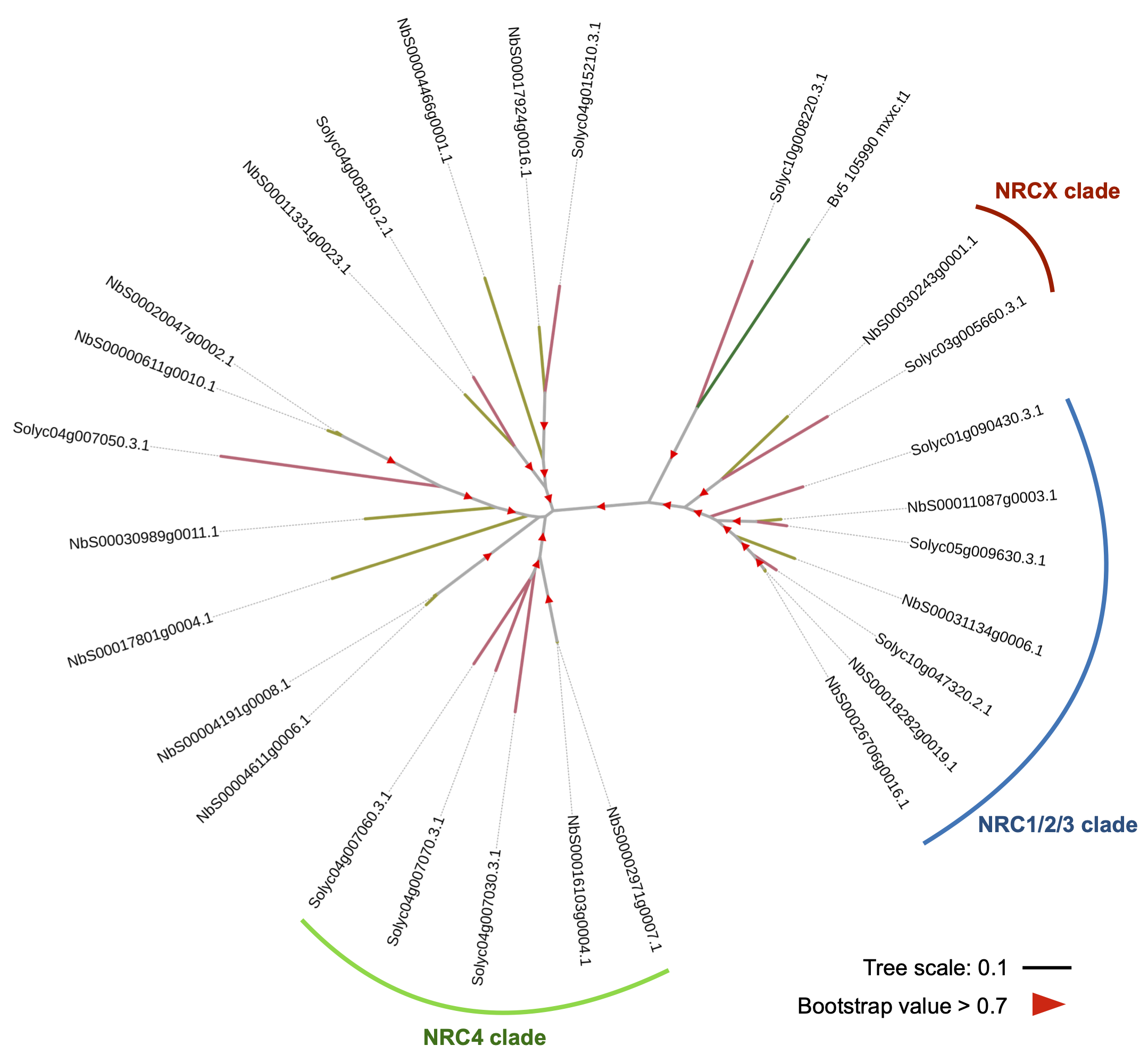

Supplement: S3 Fig — (TIF) [file pgen.1010500.s003.tif]

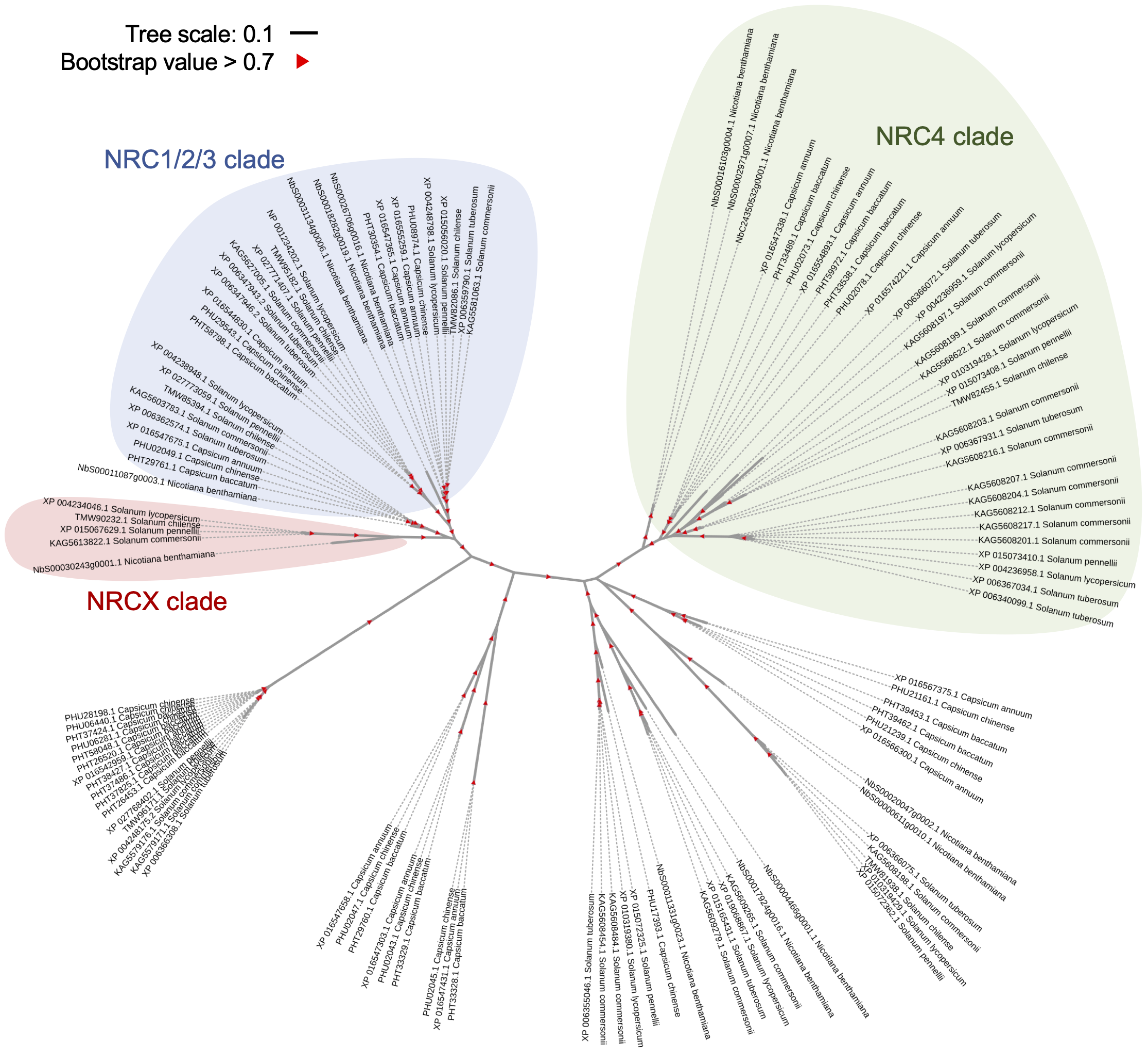

Supplement: S4 Fig — NRC-helper (NRC-H) proteins identified from Nicotiana benthamiana, Capsicum annuum, Capsicum chinense, Capsicum baccatum, Solanum commersonii, Solanum tuberosum, Solanum lycopersicum, Solanum pennellii and Solanum chilense were used for the MAFFT multiple alignment and phylogenetic analysis. The phylogenetic tree was constructed with the NB-ARC domain sequences in RAxML version 8.2.12 by the maximum likelihood method. NRCX, NRC1/2/3 and NRC4 subclades are labelled with different colour ranges. Red arrow heads indicate bootstrap support > 0.7. The scale bars indicate the evolutionary distance in amino acid substitution per site. (TIF) [file pgen.1010500.s004.tif]

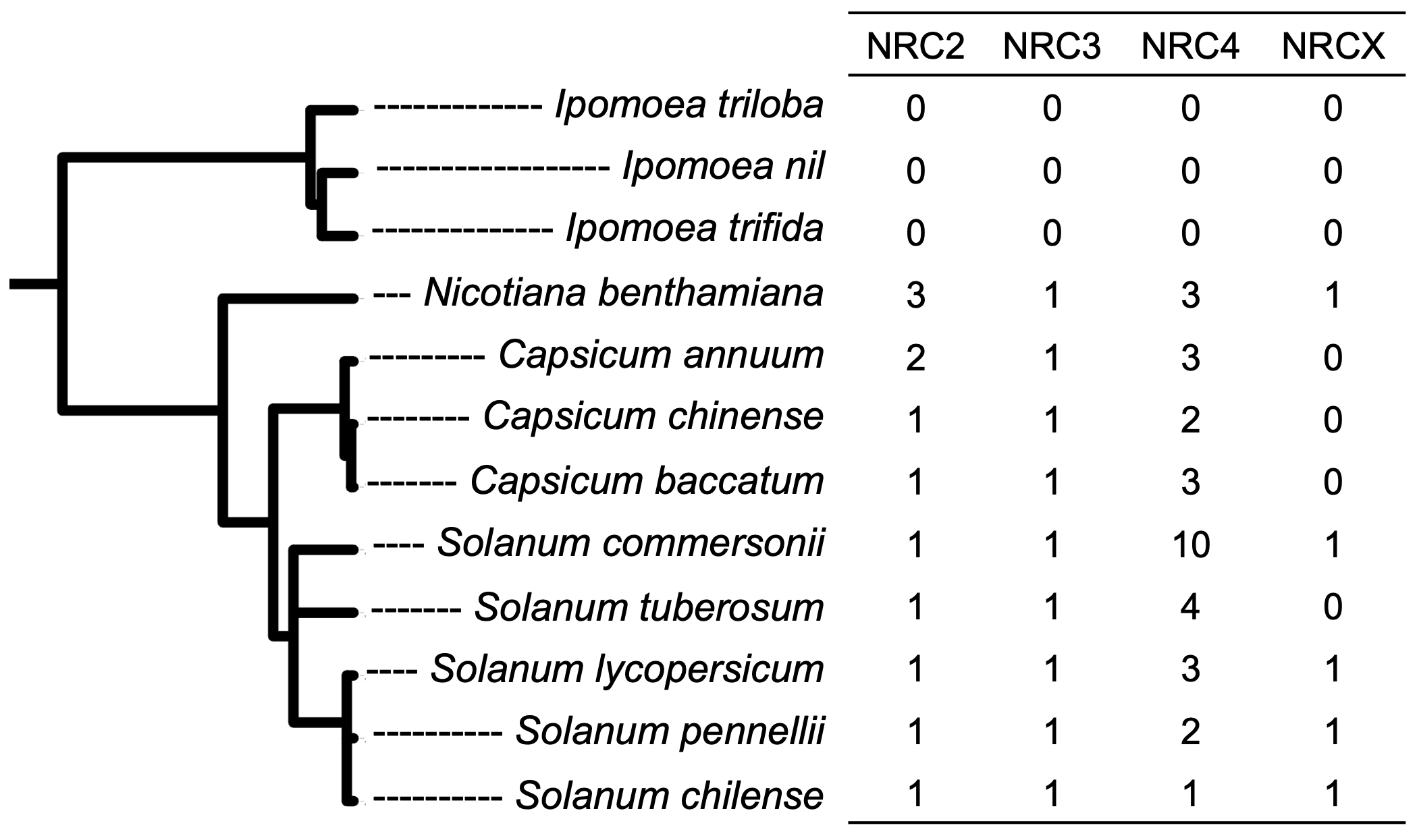

Supplement: S5 Fig — The number of ortholog genes were counted based on NRC-H phylogeny in S4 Fig. (TIF) [file pgen.1010500.s005.tif]

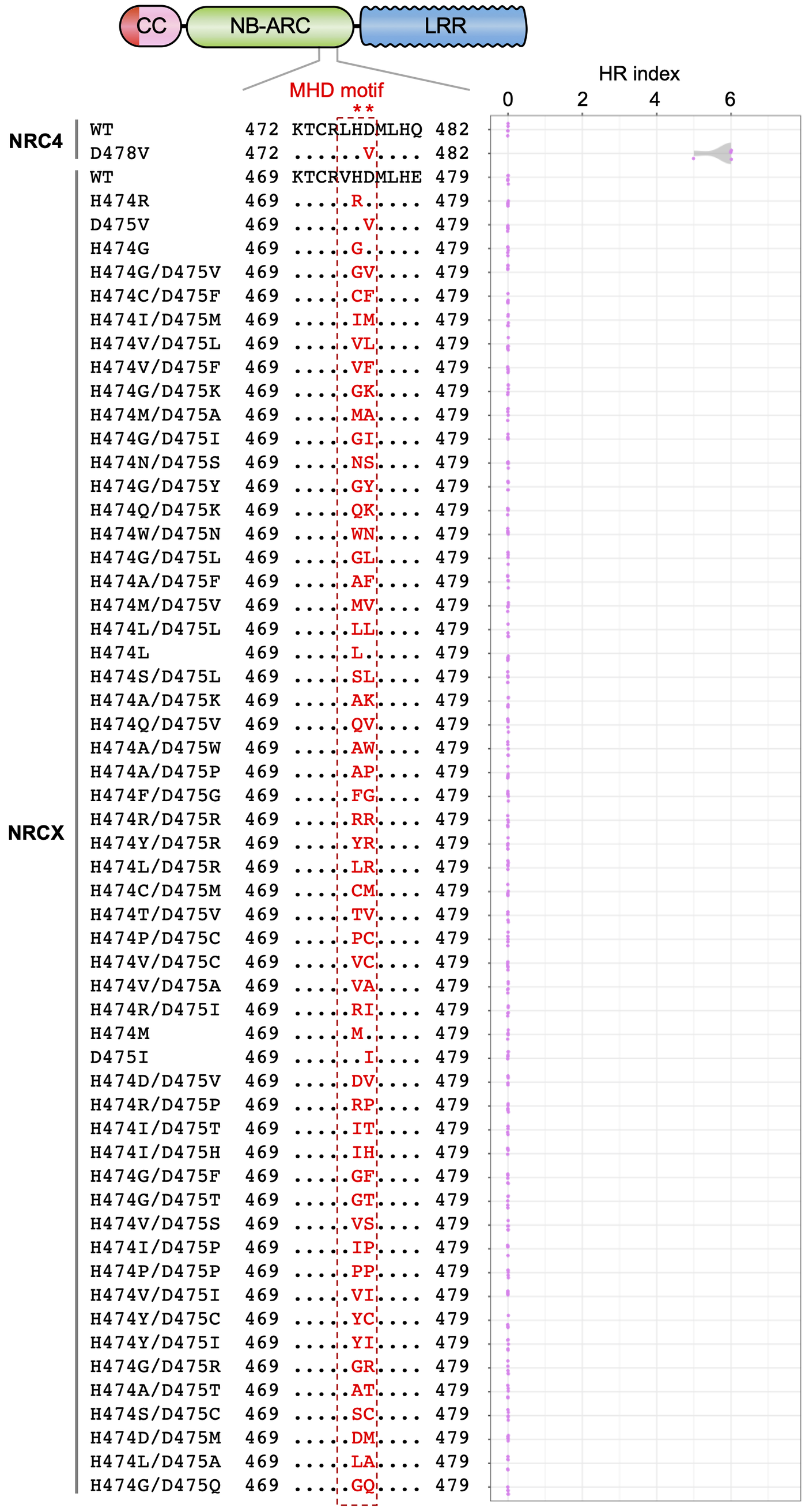

Supplement: S6 Fig — Cell death phenotypes were scored at an HR index at 5 days after agroinfiltration to express NRC4WT, NRCXWT and the MHD mutants in N. benthamiana leaves. Quantification data are from 5 independent biological replicates. (TIF) [file pgen.1010500.s006.tif]

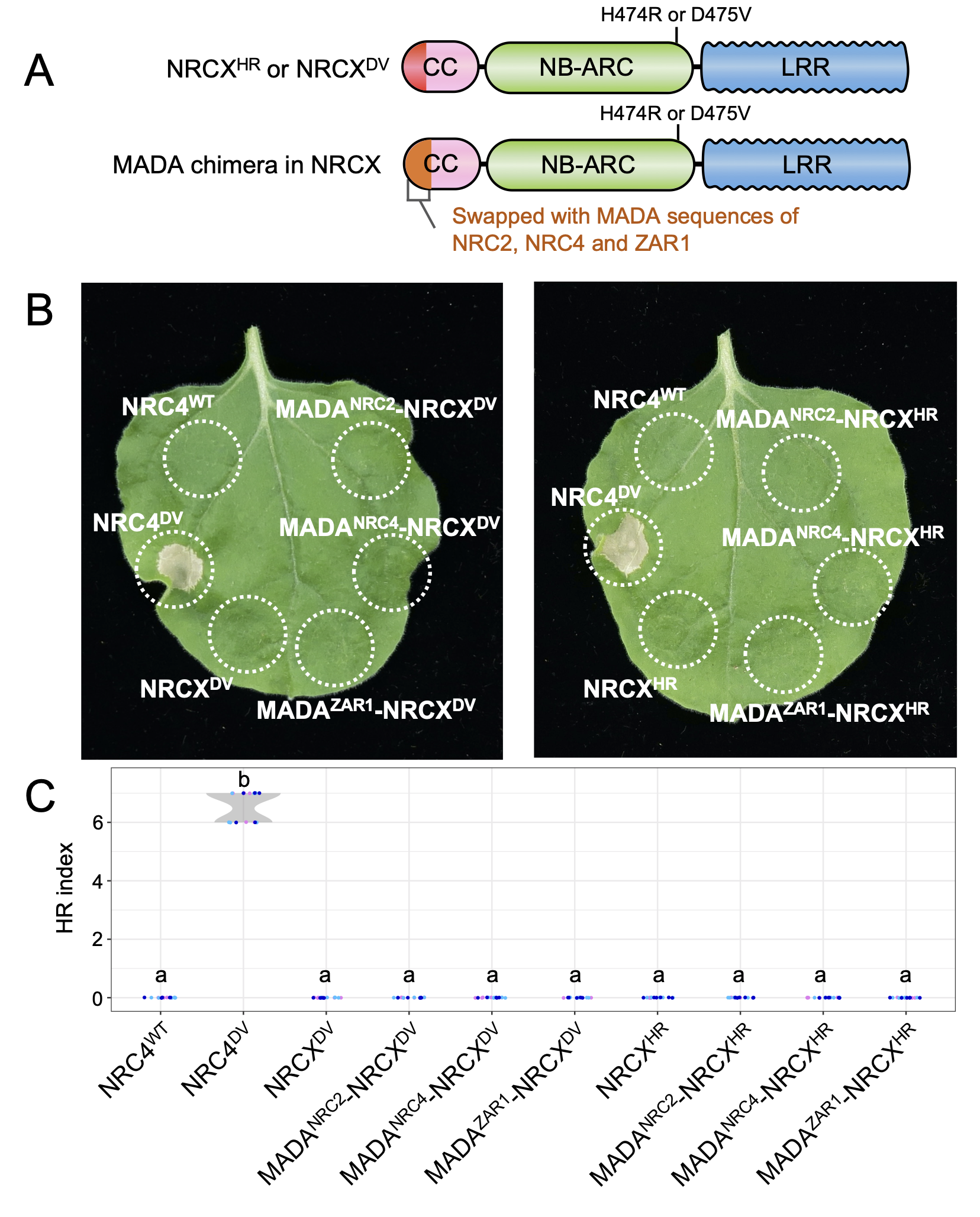

Supplement: S7 Fig — (A) Schematic representation of NRCX MADA motif chimeras. The first 17 amino acid region of NRC2, NRC4 and ZAR1 was swapped into the NRCX MHD motif mutants (NRCXHR and NRCXDV), resulting in the NRCX chimeras with MADA sequences originated from other MADA-CC-NLRs. (B) Cell death phenotypes induced by NRC4WT, NRC4DV and the NRCX chimeras. NRC4WT-6xHA, NRC4DV-6xHA and the NRCX chimeras were expressed in N. benthamiana leaves by agroinfiltration. Photographs were taken at 5 days after the agroinfiltration. (C) Violin plots showing cell death intensity scored as an HR index based on 16 or 17 different replicates in three independent experiments. Statistical differences among the samples were analyzed with Tukey’s honest significance difference (HSD) test (p<0.01). (TIF) [file pgen.1010500.s007.tif]

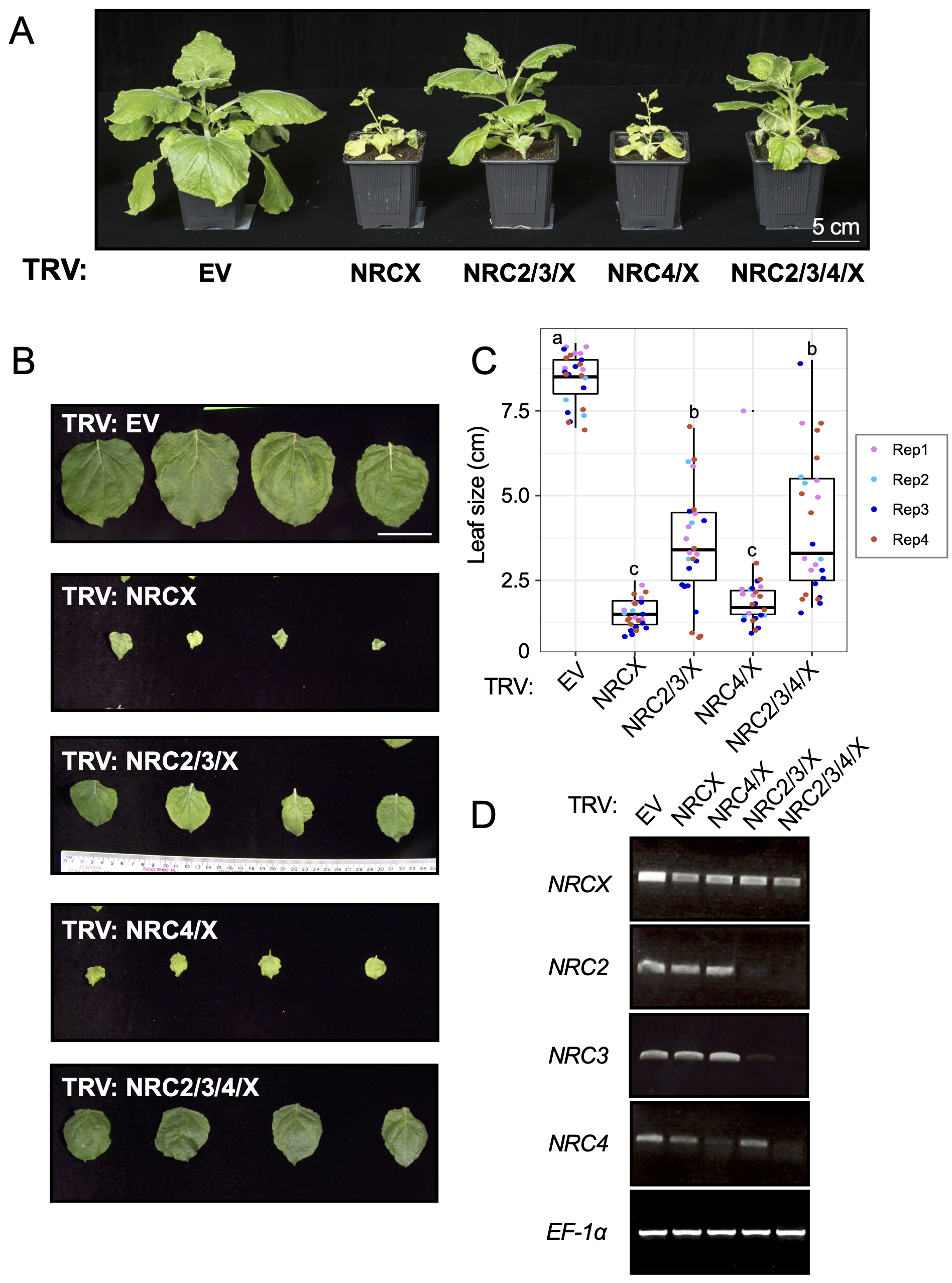

Supplement: S8 Fig — (A) The morphology of 6-week-old NRCX-, NRC2/3/X-, NRC4/X- and NRC2/3/4/X-silenced N. benthamiana plants. 2-week-old N. benthamiana plants were infiltrated with Agrobacterium strains carrying VIGS constructs, and photographs were taken 4 weeks after the agroinfiltration. TRV empty vector (TRV:EV) was used as a negative control. (B, C) Quantification of the leaf size. One leaf per each plant was harvested from the same position (the 5th leaf from cotyledons) and was used for measuring the leaf diameter. Data was obtained from 25 different VIGS plants in four independent experiments. Statistical differences among the samples were analyzed with Tukey’s HSD test (p<0.01). Scale bars = 5 cm. (D) Specific gene silencing of NRCX or multiple NRC genes in TRV:NRC-infected plants. Leaf samples were collected for RNA extraction at 3 weeks after agroinfiltration expressing VIGS constructs. The expression of NRCX and other NRC genes were analyzed in semi-quantitative RT-PCR using specific primer sets. Elongation factor 1α (EF-1α) was used as an internal control. Scale bars = 5 cm. (TIF) [file pgen.1010500.s008.tif]

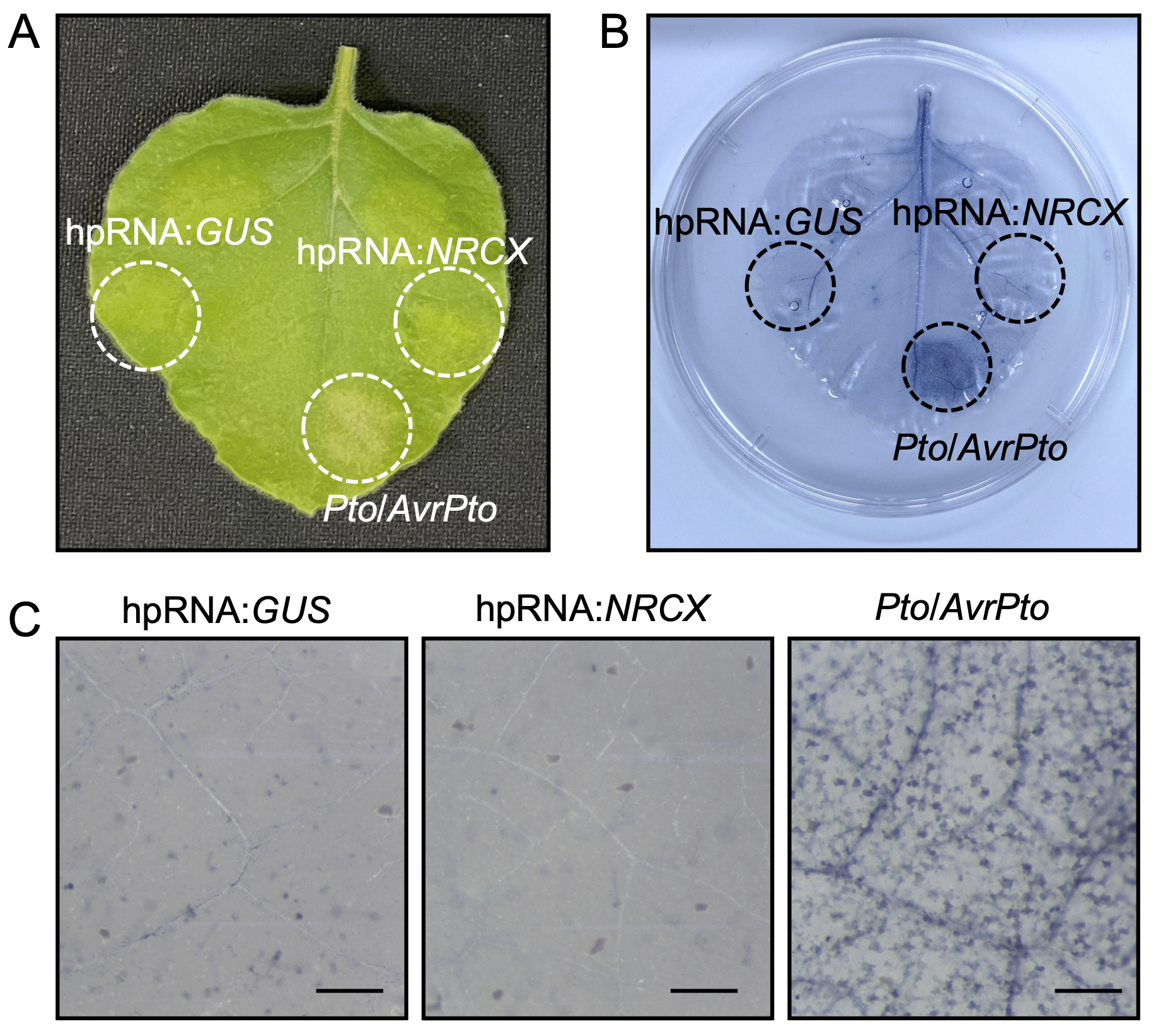

Supplement: S9 Fig — (A) Macroscopic cell death phenotype after expressing hpRNA:GUS, hpRNA:NRCX or Pto/AvrPto by agroinfiltration. Photograph was taken at 5 days after the agroinfiltration. (B) Cell death was detected by trypan blue staining at 5 days after the agroinfiltration. (C) Microscopic cell death phenotype. Dead cells were stained by trypan blue. Images describe representative data of 8 replicates from 2 independent experiments. Scale bars are 300 μm. (TIF) [file pgen.1010500.s009.tif]

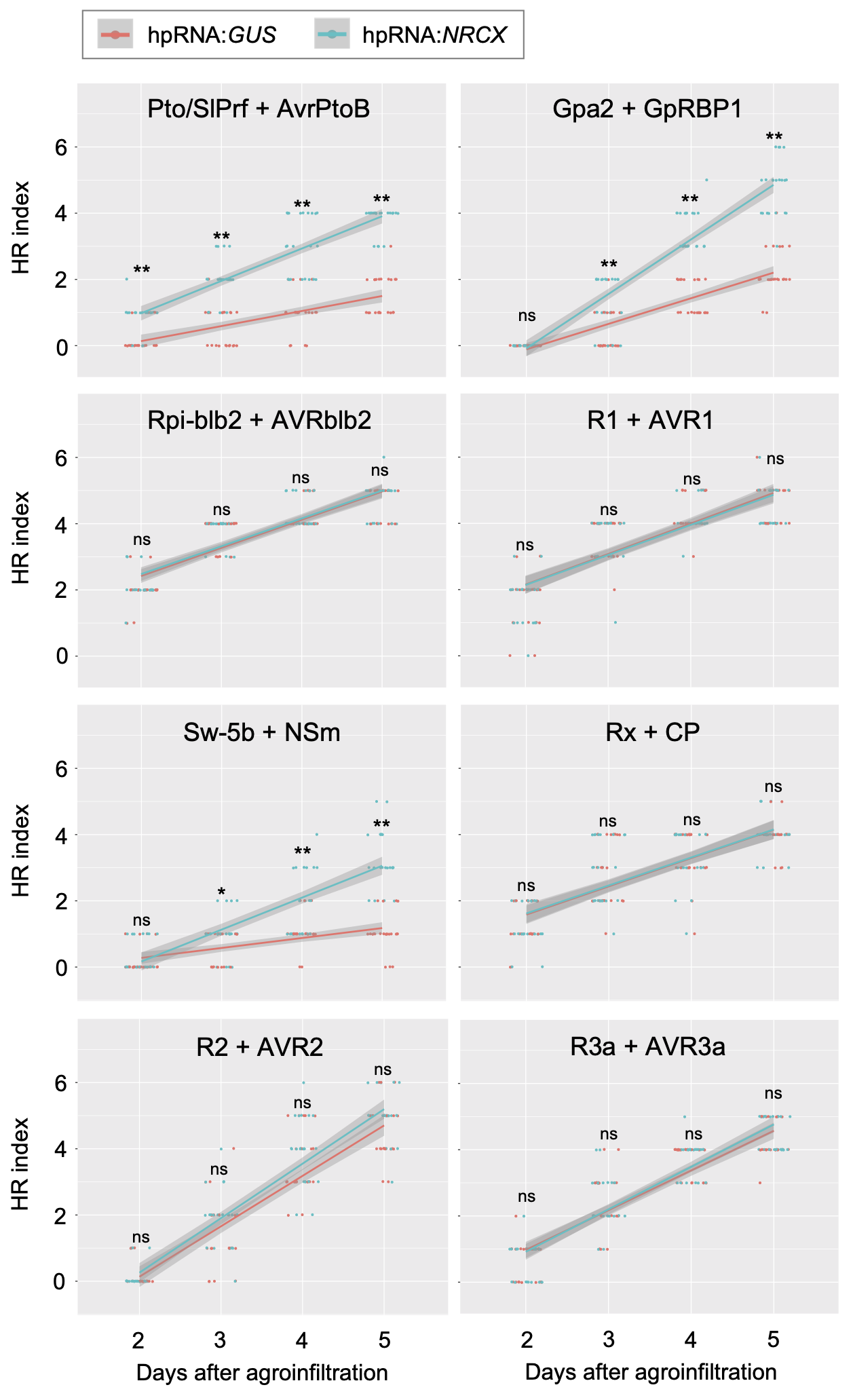

Supplement: S10 Fig — Cell death intensity was scored at 2–5 days after the agroinfiltration as described in Fig 6. Data at 5 days after agroinfiltration is the same with Fig 6B. The HR index plots are based on three independent experiments. Asterisks indicate statistically significant differences with t test (*p<0.05 and **p<0.01). Pink and blue line plots indicate mean values of hpRNA:GUS and hpRNA:NRCX samples at each time point. (TIF) [file pgen.1010500.s010.tif]

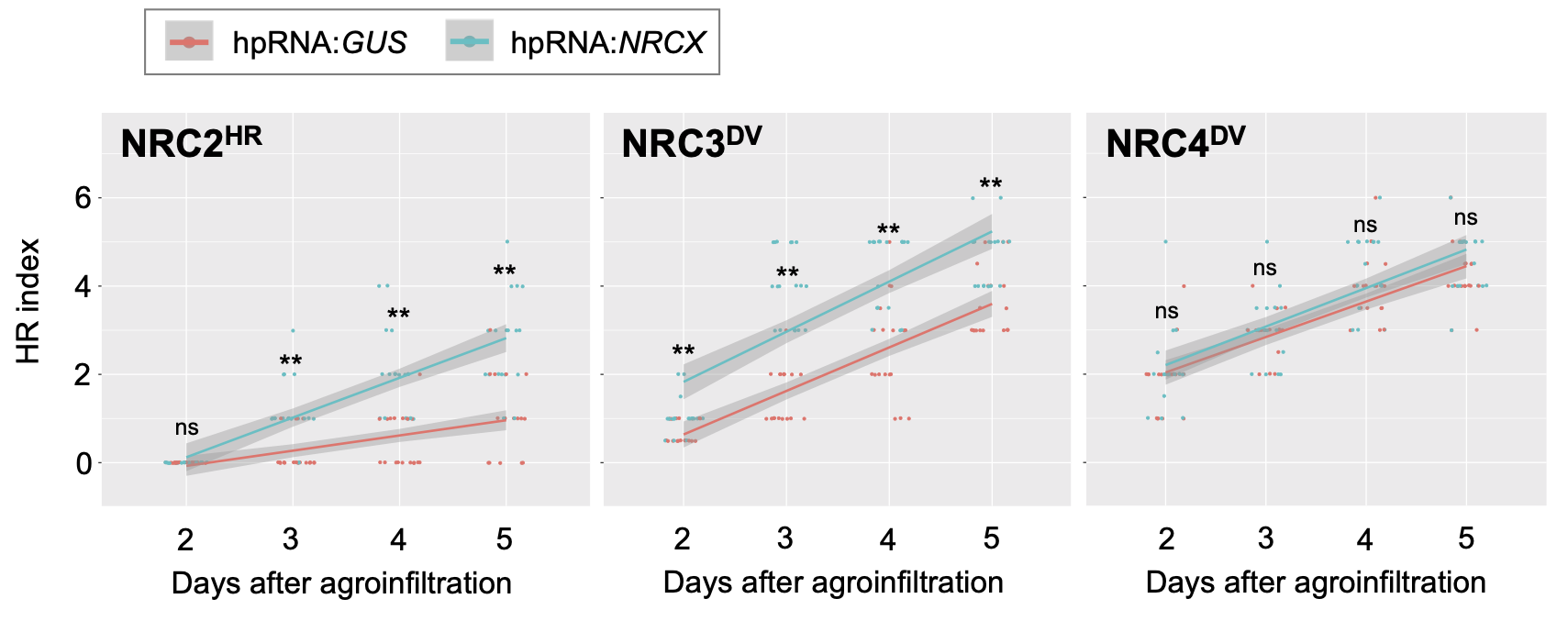

Supplement: S11 Fig — Cell death intensity was scored at 2–5 days after the agroinfiltration as described in Fig 6. The HR index plots are based on three independent experiments. Asterisks indicate statistically significant differences with t test (**p<0.01). Pink and blue line plots indicate mean values of hpRNA:GUS and hpRNA:NRCX samples at each time point. (TIF) [file pgen.1010500.s011.tif]

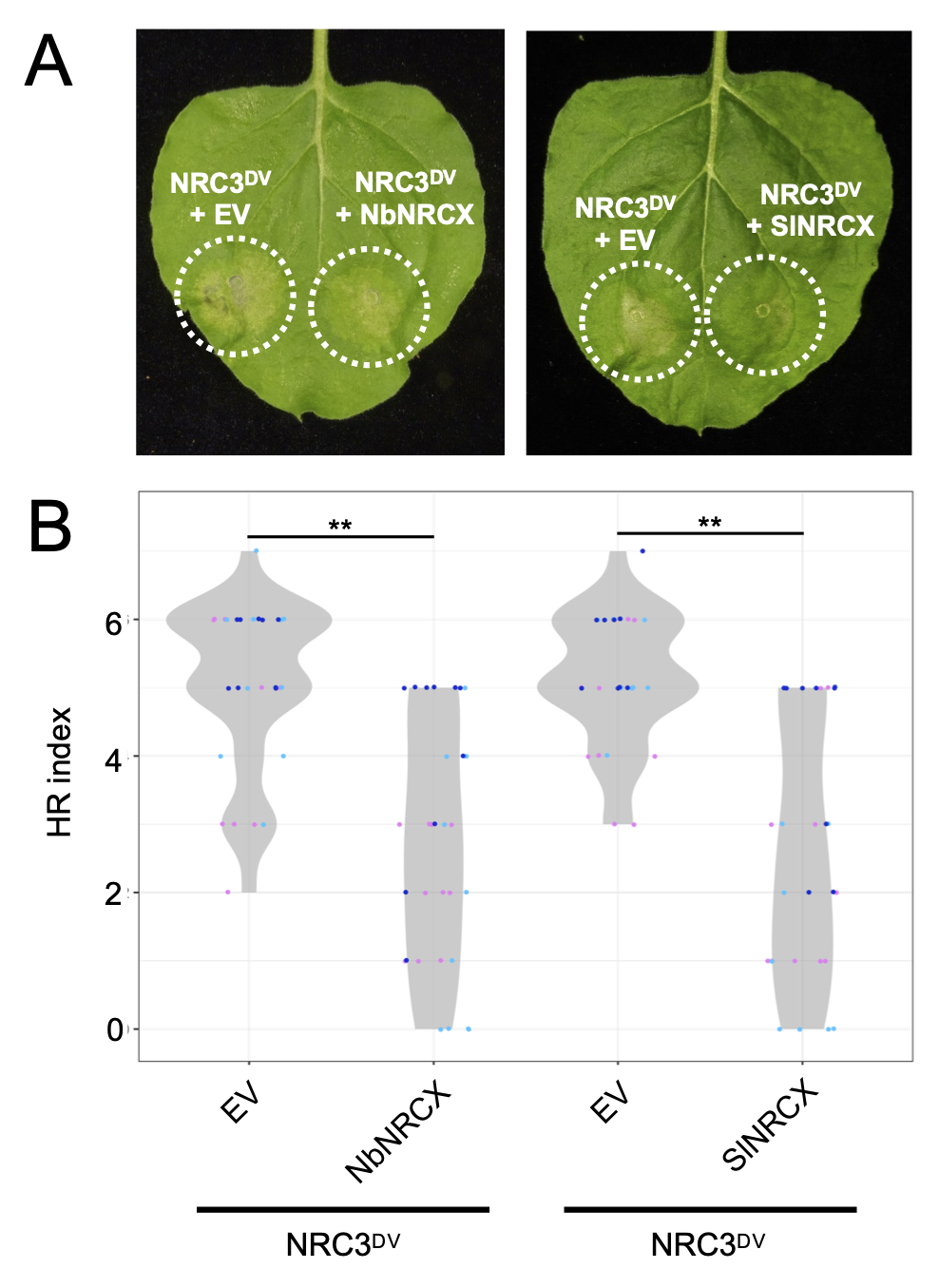

Supplement: S12 Fig — (A) Photo of representative N. benthamiana leaves showing autoactive cell death after co-expression of empty vector (EV; control) and wild-type NRCX with NRC3DV. Photographs were taken at 4 days after agroinfiltration. (B) Violin plots showing cell death intensity scored as an HR index at 4 days after the agroinfiltration. The HR index plots are based on 27 to 30 different replicates in three independent experiments. Asterisks indicate statistically significant differences with t test (**p<0.01). (TIF) [file pgen.1010500.s012.tif]
