# Supplementary material for: An atypical NLR protein modulates the NRC immune receptor network in Nicotiana benthamiana
Source: PLoS Genet. 2023 Jan 19;19(1):e1010500. doi: 10.1371/journal.pgen.1010500 (PMC9851556; doi:10.1371/journal.pgen.1010500)
Supplement: S3 Table — (PPTX) [file pgen.1010500.s015.pptx]

## Slide 1
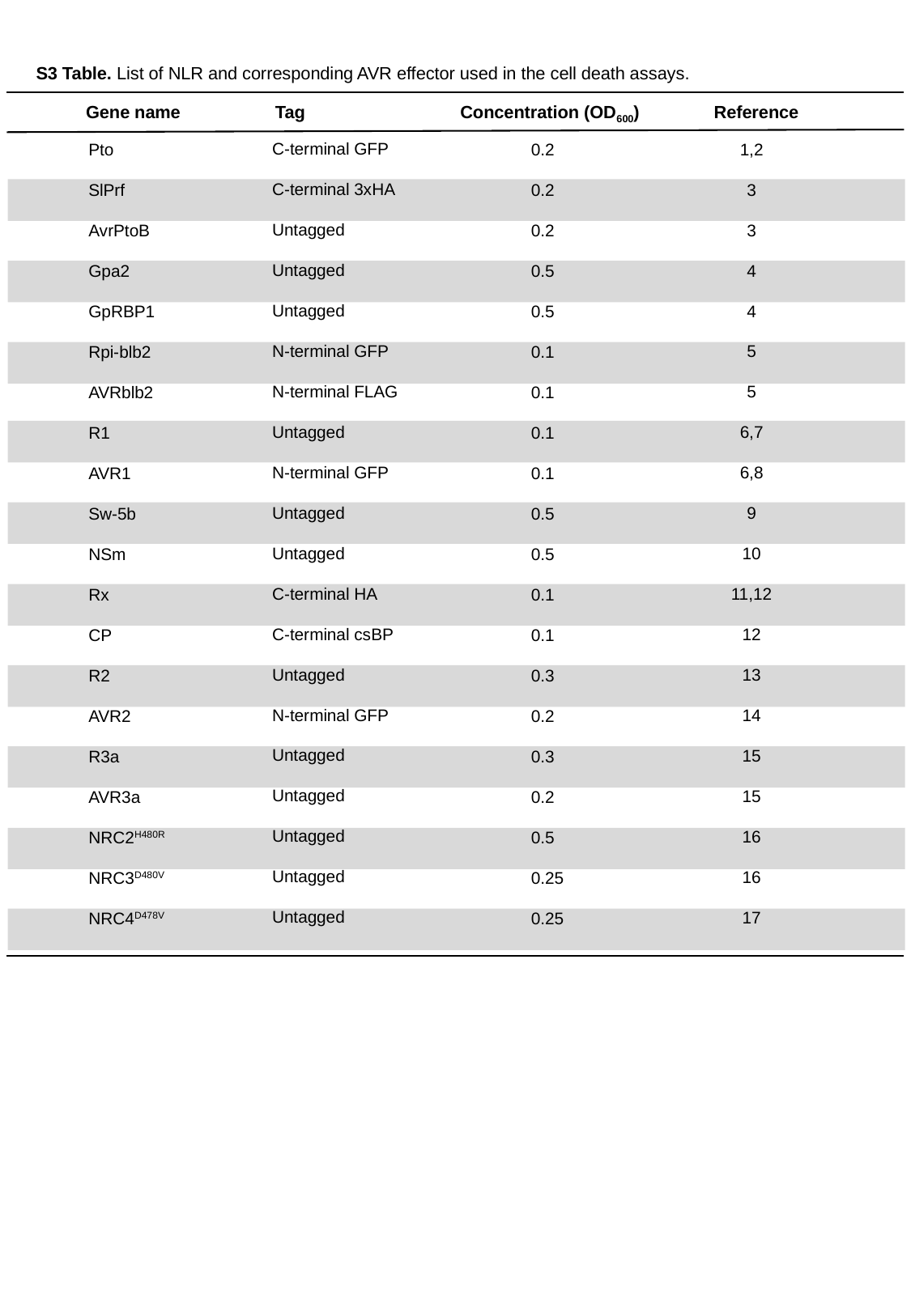

S3 Table. List of NLR and corresponding AVR effector used in the cell death assays.
Gene name
Tag
Concentration (OD600)
Reference
C-terminal GFP
C-terminal 3xHA
Untagged
Untagged
Untagged
N-terminal GFP
N-terminal FLAG
Untagged
N-terminal GFP
Untagged
Untagged
C-terminal HA
C-terminal csBP
Untagged
N-terminal GFP
Untagged
Untagged
Untagged
Untagged
Untagged
1,2
3
3
4
4
5
5
6,7
6,8
9
10
11,12
12
13
14
15
15
16
16
17
Pto
SlPrf
AvrPtoB
Gpa2
GpRBP1
Rpi-blb2
AVRblb2
R1
AVR1
Sw-5b
NSm
Rx
CP
R2
AVR2
R3a
AVR3a
NRC2H480R
NRC3D480V
NRC4D478V
0.2
0.2
0.2
0.5
0.5
0.1
0.1
0.1
0.1
0.5
0.5
0.1
0.1
0.3
0.2
0.3
0.2
0.5
0.25
0.25

## Slide 2
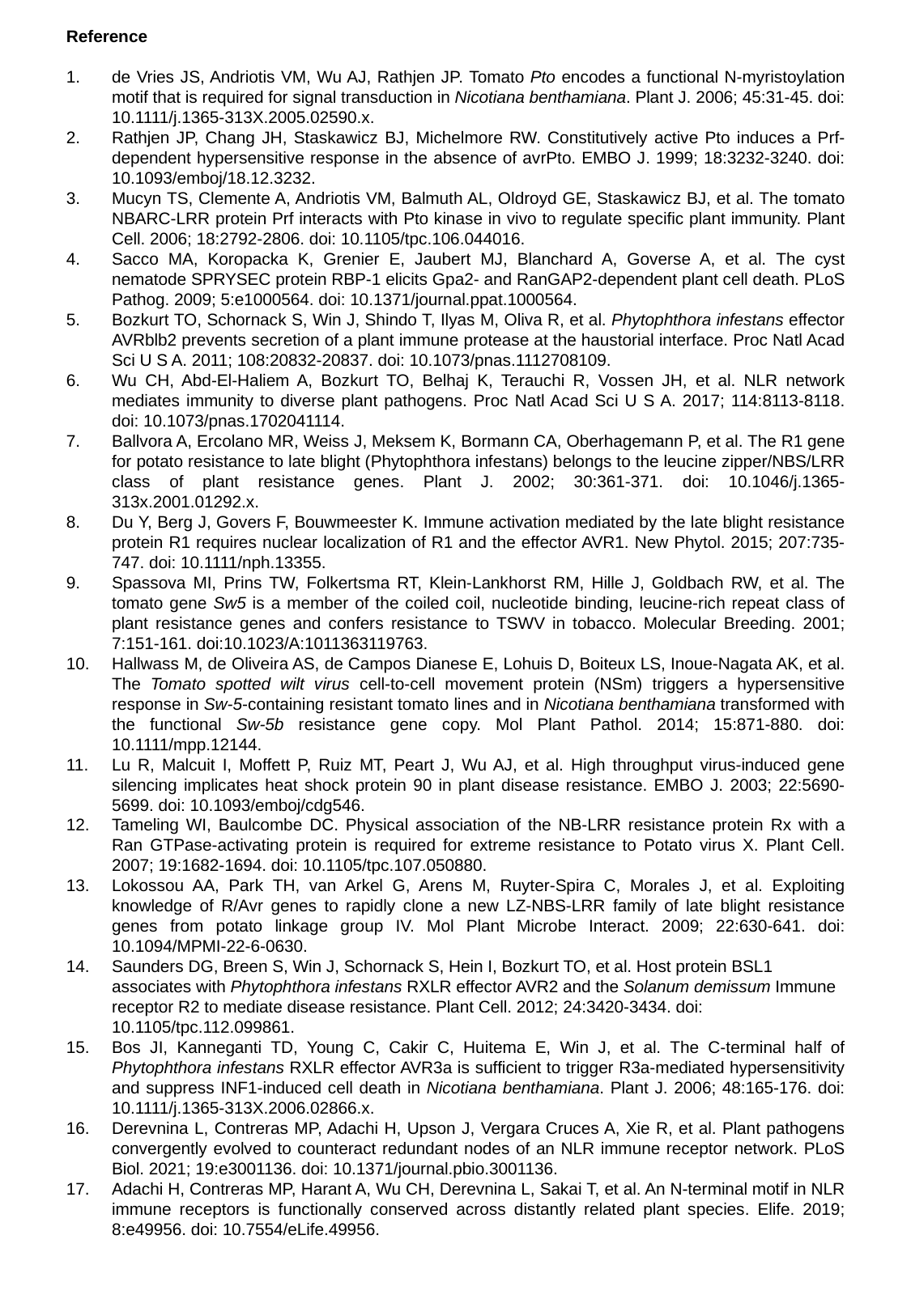

Reference
de Vries JS, Andriotis VM, Wu AJ, Rathjen JP. Tomato Pto encodes a functional N-myristoylation motif that is required for signal transduction in Nicotiana benthamiana. Plant J. 2006; 45:31-45. doi: 10.1111/j.1365-313X.2005.02590.x.
Rathjen JP, Chang JH, Staskawicz BJ, Michelmore RW. Constitutively active Pto induces a Prf-dependent hypersensitive response in the absence of avrPto. EMBO J. 1999; 18:3232-3240. doi: 10.1093/emboj/18.12.3232.
Mucyn TS, Clemente A, Andriotis VM, Balmuth AL, Oldroyd GE, Staskawicz BJ, et al. The tomato NBARC-LRR protein Prf interacts with Pto kinase in vivo to regulate specific plant immunity. Plant Cell. 2006; 18:2792-2806. doi: 10.1105/tpc.106.044016.
Sacco MA, Koropacka K, Grenier E, Jaubert MJ, Blanchard A, Goverse A, et al. The cyst nematode SPRYSEC protein RBP-1 elicits Gpa2- and RanGAP2-dependent plant cell death. PLoS Pathog. 2009; 5:e1000564. doi: 10.1371/journal.ppat.1000564.
Bozkurt TO, Schornack S, Win J, Shindo T, Ilyas M, Oliva R, et al. Phytophthora infestans effector AVRblb2 prevents secretion of a plant immune protease at the haustorial interface. Proc Natl Acad Sci U S A. 2011; 108:20832-20837. doi: 10.1073/pnas.1112708109.
Wu CH, Abd-El-Haliem A, Bozkurt TO, Belhaj K, Terauchi R, Vossen JH, et al. NLR network mediates immunity to diverse plant pathogens. Proc Natl Acad Sci U S A. 2017; 114:8113-8118. doi: 10.1073/pnas.1702041114.
Ballvora A, Ercolano MR, Weiss J, Meksem K, Bormann CA, Oberhagemann P, et al. The R1 gene for potato resistance to late blight (Phytophthora infestans) belongs to the leucine zipper/NBS/LRR class of plant resistance genes. Plant J. 2002; 30:361-371. doi: 10.1046/j.1365-313x.2001.01292.x.
Du Y, Berg J, Govers F, Bouwmeester K. Immune activation mediated by the late blight resistance protein R1 requires nuclear localization of R1 and the effector AVR1. New Phytol. 2015; 207:735-747. doi: 10.1111/nph.13355.
Spassova MI, Prins TW, Folkertsma RT, Klein-Lankhorst RM, Hille J, Goldbach RW, et al. The tomato gene Sw5 is a member of the coiled coil, nucleotide binding, leucine-rich repeat class of plant resistance genes and confers resistance to TSWV in tobacco. Molecular Breeding. 2001; 7:151-161. doi:10.1023/A:1011363119763.
Hallwass M, de Oliveira AS, de Campos Dianese E, Lohuis D, Boiteux LS, Inoue-Nagata AK, et al. The Tomato spotted wilt virus cell-to-cell movement protein (NSm) triggers a hypersensitive response in Sw-5-containing resistant tomato lines and in Nicotiana benthamiana transformed with the functional Sw-5b resistance gene copy. Mol Plant Pathol. 2014; 15:871-880. doi: 10.1111/mpp.12144.
Lu R, Malcuit I, Moffett P, Ruiz MT, Peart J, Wu AJ, et al. High throughput virus-induced gene silencing implicates heat shock protein 90 in plant disease resistance. EMBO J. 2003; 22:5690-5699. doi: 10.1093/emboj/cdg546.
Tameling WI, Baulcombe DC. Physical association of the NB-LRR resistance protein Rx with a Ran GTPase-activating protein is required for extreme resistance to Potato virus X. Plant Cell. 2007; 19:1682-1694. doi: 10.1105/tpc.107.050880.
Lokossou AA, Park TH, van Arkel G, Arens M, Ruyter-Spira C, Morales J, et al. Exploiting knowledge of R/Avr genes to rapidly clone a new LZ-NBS-LRR family of late blight resistance genes from potato linkage group IV. Mol Plant Microbe Interact. 2009; 22:630-641. doi: 10.1094/MPMI-22-6-0630.
Saunders DG, Breen S, Win J, Schornack S, Hein I, Bozkurt TO, et al. Host protein BSL1 associates with Phytophthora infestans RXLR effector AVR2 and the Solanum demissum Immune receptor R2 to mediate disease resistance. Plant Cell. 2012; 24:3420-3434. doi: 10.1105/tpc.112.099861.
Bos JI, Kanneganti TD, Young C, Cakir C, Huitema E, Win J, et al. The C-terminal half of Phytophthora infestans RXLR effector AVR3a is sufficient to trigger R3a-mediated hypersensitivity and suppress INF1-induced cell death in Nicotiana benthamiana. Plant J. 2006; 48:165-176. doi: 10.1111/j.1365-313X.2006.02866.x.
Derevnina L, Contreras MP, Adachi H, Upson J, Vergara Cruces A, Xie R, et al. Plant pathogens convergently evolved to counteract redundant nodes of an NLR immune receptor network. PLoS Biol. 2021; 19:e3001136. doi: 10.1371/journal.pbio.3001136.
Adachi H, Contreras MP, Harant A, Wu CH, Derevnina L, Sakai T, et al. An N-terminal motif in NLR immune receptors is functionally conserved across distantly related plant species. Elife. 2019; 8:e49956. doi: 10.7554/eLife.49956.
