# Supplementary material for: An atypical NLR protein modulates the NRC immune receptor network in Nicotiana benthamiana
Source: PLoS Genet. 2023 Jan 19;19(1):e1010500. doi: 10.1371/journal.pgen.1010500 (PMC9851556; doi:10.1371/journal.pgen.1010500)
Supplement: Attachment — Submitted filename: Response to reviewer comments_submitted.docx [file pgen.1010500.s029.docx]

Dear Dr. Gitta Coaker and Dr. Claudia Köhler,

Thank you very much for your evaluation of our manuscript " An atypical NLR protein modulates the NRC immune receptor network in *Nicotiana benthamiana* " for consideration as a research article at PLOS Genetics.

We appreciate the overall positive feedback of the independent reviewers. We have endeavored in this revision to address as much as possible the pertinent points they raised. Please see the point-by-point 'Response to Reviewers' attached to this file.

We hope that we have addressed many of the issues that were raised and we look forward to your response.

Best regards,

Sophien Kamoun

Dear Dr Kamoun,

Thank you very much for submitting your Research Article entitled 'An atypical NLR protein modulates the NRC immune receptor network' to PLOS Genetics.

The manuscript was fully evaluated at the editorial level and by independent peer reviewers. The reviewers appreciated the attention to an important problem, but raised some substantial concerns about the current manuscript. Based on the reviews, we will not be able to accept this version of the manuscript, but we would be willing to review a much-revised version. We cannot, of course, promise publication at that time.

Should you decide to revise the manuscript for further consideration here, your revisions should address the specific points made by each reviewer. We will also require a detailed list of your responses to the review comments and a description of the changes you have made in the manuscript.

If you decide to revise the manuscript for further consideration at PLOS Genetics, please aim to resubmit within the next 60 days, unless it will take extra time to address the concerns of the reviewers, in which case we would appreciate an expected resubmission date by email to plosgenetics@plos.org.

If present, accompanying reviewer attachments are included with this email; please notify the journal office if any appear to be missing. They will also be available for download from the link below. You can use this link to log into the system when you are ready to submit a revised version, having first consulted our Submission Checklist.

To enhance the reproducibility of your results, we recommend that you deposit your laboratory protocols in protocols.io, where a protocol can be assigned its own identifier (DOI) such that it can be cited independently in the future. Additionally, PLOS ONE offers an option to publish peer-reviewed clinical study protocols. Read more information on sharing protocols at https://plos.org/protocols?utm_medium=editorial-email&utm_source=authorletters&utm_campaign=protocols

Please be aware that our data availability policy requires that all numerical data underlying graphs or summary statistics are included with the submission, and you will need to provide this upon resubmission if not already present. In addition, we do not permit the inclusion of phrases such as "data not shown" or "unpublished results" in manuscripts. All points should be backed up by data provided with the submission.

While revising your submission, please upload your figure files to the Preflight Analysis and Conversion Engine (PACE) digital diagnostic tool. PACE helps ensure that figures meet PLOS requirements. To use PACE, you must first register as a user. Then, login and navigate to the UPLOAD tab, where you will find detailed instructions on how to use the tool. If you encounter any issues or have any questions when using PACE, please email us at figures@plos.org.

PLOS has incorporated Similarity Check, powered by iThenticate, into its journal-wide submission system in order to screen submitted content for originality before publication. Each PLOS journal undertakes screening on a proportion of submitted articles. You will be contacted if needed following the screening process.

To resubmit, use the link below and 'Revise Submission' in the 'Submissions Needing Revision' folder.

Please do not hesitate to contact us if you have any concerns or questions.

Yours sincerely,

Gitta Coaker, PhD

Associate Editor

PLOS Genetics

Claudia Köhler

Section Editor: Plant Genetics

PLOS Genetics

Editor comments (Gitta Coaker)

All reviewers appreciated the subject matter of the paper and generally felt experiments were well-done, but also suggested several new experiments. These experiments differed between reviewers. Given the general positive comments, I do not think major new experimentation to address all major reviewer comments should be done. Rather, I have provided some guidance below with respect to major comments (minor comments can be textually addressed).

Thank you very much for your evaluation and comments. We revised our manuscript and prepared point-by-point responses to editor and reviewer comments.

Deposit RNAseq data to a public database

We deposited all RNAseq data to public database. Accession numbers are listed in Data availability section.

“RNA-seq raw reads used for transcriptomic analyses in this study have been deposited under the BioProject accessions: TRV:*GUS*_leaf and TRV:*NRCX*_leaf (PRJEB55392), and five-week old wild-type *N. benthamiana*_leaf, root and flower/bud (PRJEB55516).”

Consider if it is possible to investigate, or at minimum futher discuss, potential reasons for NRCX MADA’s inability to trigger cell death when fused to an autoactive variant, including misfolding.

Reviewer 4 has proposed some informative experiments: “Have the authors tried to see if NRCX or NRCX DV when its alpha 1 helix is swapped with the alpha 1 helix of NRC4 is able to cause cell death? This would give insights whether NRCX would in general be able to function in cell death or whether other constraints are in work. Would it be possible to complement the severe dwarf phenotype of the NRCX silenced plants with a construct that would allow expression of a silencing resistant NRCX version?”

Thank you very much for your suggestion. We conducted a suggested experiment by Reviewer 4. We generated MADA chimera constructs in the NRCX^DV^ and NRCX^HR^ MHD mutant backgrounds by using NRC2, NRC4 and ZAR1 MADA sequences. Although the NRCX chimeras have functional MADA sequences derived from the other MADA-CC-NLRs, the NRCX chimeras did not cause visible autoactive cell death response when expressed in *N. benthamiana* leaves. Therefore, we conclude that in addition to the non-functional MADA region, there is other constrain(s) in NRCX protein, thereby losing autoactive cell death activity unlike other helper NRCs. We discussed our new data in result and discussion:

“We also performed the opposite motif swap to test whether functional MADA sequences confer gain of cell death activity to NRCX. To this end, we produced MADA chimera constructs in the NRCX^DV^ and NRCX^HR^ MHD motif mutant backgrounds (S7A Fig). Although the N-terminal MADA sequences from NRC2 and ZAR1 are functional in NRC4^DV^ (Fig 4) [24], MADA^NRC2^-NRCX^DV^, MADA^ZAR1^-NRCX^DV^, MADA^NRC2^-NRCX^HR^ and MADA^ZAR1^-NRCX^HR^ did not induce any visible cell death when expressed in *N. benthamiana* leaves (S7B and S7C Fig). We further tested NRCX^DV^ and NRCX^HR^ chimera constructs with NRC4 MADA sequence (S7A Fig). Neither MADA^NRC4^-NRCX^DV^ nor MADA^NRC4^-NRCX^HR^ caused visible cell death response in *N. benthamiana* leaves (S7B and S7C Fig). These results suggest that in addition to the non-functional MADA region, there is other constrain(s) in NRCX protein that led to the incapacity to cause autoactive cell death unlike other helper NRCs.”

“NRCX clusters within the NRC-H subclade, which is populated with proteins with a MADA-CC-NLR architecture (Fig 2). However, the N-terminal MADA motif of NRCX was not functional when swapped into NRC4 (Fig 4), compared to other similar swaps we previously tested [10,24,66]. We conclude that the MADA motif of NRCX has degenerated from the canonical sequence present in the typical NRC helper NLRs and lost the capacity to execute the hypersensitive cell death response. In fact, a polymorphism in the NRCX MADA motif region, Thr-4, is unique to this protein among NRC-H (Fig 2). Moreover, given that functional MADA sequences did not confer cell death activity to NRCX (S7 Fig), NRCX may have become inactive to trigger cell death response by other mutation(s). Therefore, loss of cell death executor activity in NRCX might represent another path in the evolution of networked NLRs besides sensor and helper NLRs, as in the NLR functional specialization “use-it-or-lose-it” model described by Adachi et al. [24] and Kamoun [67]. We propose that NRCX has also functionally specialized into an atypical NLR protein that operates as an NLR modulator.”

To perform complementation assay using silencing resistant NRCX version, we first need to make stable transgenic *N. benthamiana* plants expression the *NRCX* variant gene and then conduct systemic gene silencing experiment of *NRCX*. Considering that the suggested complementation experiment will take a lot of time, we would like to address this question in future.

I do not think it is necessary to generate a knockout in nrcx as it will likely be lethal (but provide more data on specificity - can be in silico data on off targets).

Thank you very much for your suggestion. We tried CRISPR/Cas9-mediated gene editing by targeting coding region of *NRCX* gene. However, as the reviewer predicted we failed to obtain *NRCX* knockout in wild-type *N. benthamiana* plant background. In future, we will address generation of *NRCX* knockout line by using different sgRNAs and/or by using *nrc2/3*-knockout mutant lines. These experiments are complicated and require multiple generations to obtain homozygous lines and robust results.

We searched *NRCX* 446-bp sequence used for silencing experiments in Solanaceae Genomics Network VIGS tool (<https://vigs.solgenomics.net/>). This did not find out off-target candidate genes in two versions of *N. benthamiana* genome databases. We documented the *in-silico* search result and made S2 Fig to present *NRCX* sequence used for silencing experiments.

“This VIGS construct was made by using 5’ 446-bp sequence of *NRCX* gene that does not hit off-target candidate genes in Solanaceae Genomics Network VIGS Tool search (https://vigs.solgenomics.net/) using Nicotiana benthamiana v0.4.4 and Nicotiana benthamiana v1.0.1 databases (S2 Fig).”

Please examine if constitutive defense occurs in the NRCX silenced line.

Thank you very much for your suggestion. To examine whether constitutive defense activation occurs in the *NRCX* silenced plants, we extracted total RNA from leaf tissue of TRV:*NRCX* and TRV:*GUS* plants and subjected to RNA-seq analysis. Comparing to TRV:*GUS* control, in total 3162 genes were differentially expressed in TRV:*NRCX* plants. Out of 3162 genes, 1445 genes were up-regulated and 1717 genes were down-regulated. Gene Ontology (GO) analysis revealed that GO terms ‘response to wounding’ and ‘response to biotic stimulus’ are significantly enriched in up-regulated genes. Therefore, we conclude that constitutive defense response is induced in the *NRCX* silenced plants. We have this data in Fig 1C and 1D and discussed in result:

“To determine whether constitutive defense activation occurs in the dwarf plants of TRV:*NRCX*, total RNA was extracted from leaf tissue of four-week-old TRV:*NRCX* and TRV:*GUS* plants and were subjected to RNA-seq analysis. In total, 3162 genes were detected as differentially expressed genes (DEGs, TRV:*NRCX* vs TRV:*GUS*), in which 1445 and 1717 genes were up-regulated and down-regulated, respectively (Fig 1C, S1 and S2 Files). The DEGs do not include NRC-H genes such as *NRC2*, *NRC3* and *NRC4* (S1 and S2 Files). Gene Ontology (GO) analysis showed significant enrichment of GO terms ‘response to wounding’ and ‘response to biotic stimulus’ in up-regulated DEGs, while ‘cell redox homeostasis’ is enriched in down-regulated DEGs (Fig 1D). DEGs having the GO terms ‘response to wounding’ and ‘response to biotic stimulus’ include genes encoding Proteinase inhibitor I and pathogenesis related proteins (S3 File). These results suggest that constitutive defense response is induced by *NRCX* systemic silencing, thereby resulting in the dwarf phenotype in *N. benthamiana* plants.”

Also, it would be informative to determine if NRCX act as a dominant-negative helper form of NLRs (reviewer 2, also noted in reviewer 3’s comments).

Thank you very much for your suggestion. As reviewers commented, understanding how NRCX modulates the NRC2/3 network is an important question. To address this. we need to overcome technical difficulties of use of NRCX protein in biochemical and cell biology experiments. For instance, we so far faced low protein accumulation issue with NRCX protein. Such technical issue particularly affects biochemical and cell biology experiments. Our strategy is to first publish an exhaustive genetic analysis (in PLOS Genetics, a genetics-focused journal) and save the biochemical work for later. As a follow-up paper, we will improve this issue and will address mechanistic insight of NRCX-mediated regulation of NRC2 and NRC3 subnetwork.

Reviewer's Responses to Questions

Comments to the Authors:

Please note here if the review is uploaded as an attachment.

Reviewer #1:

This manuscript reports identification and characterization of the NLR NRCX that functions as a modulator of the network mediated by the helper NLRs NRC2/3 in Nicotiana benthamiana.

The authors showed that VIGS of NRCX, a paralog of NRC2/3, results in a dwarf phenotype. Then, they showed that MADA motif of NRCX is not functional for cell death-inducing activity. Consistently, mutations in MHD motif of NRCX (that would make NLR autoactive) did not cause cell death in contrast to mutations in other NRCs such as NRC4. Importantly, the dwarf phenotype caused by NRCX VIGS was dependent on NRC2/3. Suppression of NRCX enhanced cell death triggered by autoactive NRC2/3 or NLR activation which depends on NRC2/3. Consistently, overexpression of NRCX inhibited cell death triggered by autoactive NRC2/3. Finally, they showed that expression of other NLRs including NRC2 increased in PTI but not that of NRCX. Based on these results, they propose that NRCX modulates the NRC network.

This study is conceptually similar to a recent publication in Plant Cell (Wu et al in press) where they showed that NRG1c (a truncated NRG1) antagonizes immunity mediated by NRG1a and NRG1b. Nevertheless, there are important differences between two studies. In this study, NRCX and NRC2/3 are genetically unlinked while all NRG1 genes are tandemly aligned on the Arabidopsis genome and NRCX is full length NLR but NRG1c is truncated. Thus, I think that the recent Plant Cell paper does not compromises the significance of this work. An obvious weakness of this study is that the authors did not show the mechanism by which NRCX antagonizes NRC2/3. Another weakness is that the authors used only VIGS but not genetic mutation for NRCX loss of function analysis. However, I believe that these will be an important “future work” in the authors’ laboratory.

Thank you very much for your evaluation and comments. In this study, we mainly focus on identification and genetic characterization of *NRCX* in the NLR network. We hope our finding fits well to PLOS Genetics journal. In future, we aim to report mechanistic insight how NRCX modulates NRC2 and NRC3 subnetwork.

See above in response to Editor about CRISPR/Cas9-mediated gene editing of *NRCX*.

The most experiments were well performed and the manuscript is well written although I have one major suggestion and various minor suggestions that the authors might or might not consider for improving this manuscript.

(Major comments)

The incapability of NRCX MADA inducing cell death is interesting considering high amino acid sequence similarities to NRC2 and NRC4 MADA (even higher HMM score than ZAR1 MADA). No cell death triggering activity with the full length (Fig4) might be protein conformation issue but it is surprising to see that even NRCX MADA fusing autoactive NRC4 does not trigger cell death. Can the authors investigate, do AlphaFold2, or discuss more what kind of MADA has cell death inducing activity? In addition, does antagonistic activity of NRCX need NRCX MADA or not? While I think that these would increase the significance and clarity of this study, these may be beyond the scope of this study.

Thank you very much for your suggestion. Instead of doing AlphaFold2, we challenged a question why NRCX does not have cell death inducing activity by using MADA chimera constructs of NRCX^DV^ and NRCX^HR^ MHD mutants. Although the NRCX chimeras have functional MADA sequences derived from the other MADA-CC-NLRs, the NRCX chimeras did not cause visible autoactive cell death response when expressed in *N. benthamiana* leaves. Therefore, we propose that in addition to the non-functional MADA region, there is other constrain(s) in NRCX protein, thereby losing autoactive cell death activity unlike other helper NRCs. We discussed our new data:

“We also performed the opposite motif swap to test whether functional MADA sequences confer gain of cell death activity to NRCX. To this end, we produced MADA chimera constructs in the NRCX^DV^ and NRCX^HR^ MHD motif mutant backgrounds (S7A Fig). Although the N-terminal MADA sequences from NRC2 and ZAR1 are functional in NRC4^DV^ (Fig 4) [24], MADA^NRC2^-NRCX^DV^, MADA^ZAR1^-NRCX^DV^, MADA^NRC2^-NRCX^HR^ and MADA^ZAR1^-NRCX^HR^ did not induce any visible cell death when expressed in *N. benthamiana* leaves (S7B and S7C Fig). We further tested NRCX^DV^ and NRCX^HR^ chimera constructs with NRC4 MADA sequence (S7A Fig). Neither MADA^NRC4^-NRCX^DV^ nor MADA^NRC4^-NRCX^HR^ caused visible cell death response in *N. benthamiana* leaves (S7B and S7C Fig). These results suggest that in addition to the non-functional MADA region, there is other constrain(s) in NRCX protein that led to the incapacity to cause autoactive cell death unlike other helper NRCs.”

By using the MADA chimera constructs, we will address mechanistic insight how NRCX antagonizes NRC2 and NRC3 subnetwork in future.

(Minor comments)

1. I think that the authors should deposit the generated raw RNA-seq data to a public database.

Thank you for pointing this out. We deposited all RNAseq data to public database. Accession numbers are listed in Data availability section.

“RNA-seq raw reads used for transcriptomic analyses in this study have been deposited under the BioProject accessions: TRV:*GUS*_leaf and TRV:*NRCX*_leaf (PRJEB55392), and five-week old wild-type *N. benthamiana*_leaf, root and flower/bud (PRJEB55516).”

2. The authors may combine Figure S4A,C and S8A,B with Figure 5 and 6 and show them as main figures. I find them important.

We would like to keep them as separated figures. Instead, we moved Figure S8 to Figure 7.

3. The recent Wu et al Plant Cell paper (http://doi.org/10.1093/plcell/koab285) is not listed in the reference.

Thank you for pointing this out. We have updated the citation in reference.

4. Page2. “Other Arabidopsis NLRs……” NLRs are not suppressors but mutations are.

Thank you for pointing this out. We corrected the sentence as you suggested.

5. The last paragraph of Introduction. The authors may make the significance of this study clearer. There are described mechanisms that attenuate NLR networks such as by sensor NLRs, small RNAs… Considering the recent Wu et al Plant Cell, “unknown” may be too much.

Thank you for your suggestion. We rephrased the sentence:

“Yet, our knowledge of the regulatory mechanisms that attenuate such deleterious effects of NLR networks especially Solanaceae NRC networks are limited.”

6. The first two sentences of Results seem redundant with Introduction and may not necessary.

We removed the first two sentences of Results.

7. Page 4. “NRCX silencing could lead to NLR mis-regulation, thereby resulting in the dwarf phenotype in N. benthamiana plants.” This sentence may confuse readers and may be removed.

We removed this sentence as suggested.

8. Page 10. “Our finding that NRC2 and NRC3 are genetic suppressors of NRCX raises the possibility……” Mutations in NRC2 and NRC3 are genetic suppressors of phenotypes caused by NRCX silencing.

Thank you for pointing this out. We corrected the sentence.

9. Page 12. “….. all three tissues, leaf, root and flower/bud at relatively similar ratios.” I would not call these “similar ratios”.

We removed “at relatively similar ratios” from the sentence.

10. Page 12. “These transcriptome profiles suggest….” I feel this conclusion is not well supported by Fig7.

We rephrased our conclusion:

“Considering that *NRCX* is coexpressed with *NRC2* and *NRC3* in leaf, root and flower/bud, we propose that NRCX maintains NRC2/3 subnetwork homeostasis during these developmental stages.”

11. Page 12. “We conclude that following activation of…..” The authors may rewrite this.

We revised the sentence:

“We conclude that following activation of immunity in response to bacterial inoculation, *NRC2a/b* become more highly expressed than their paralog and modulator *NRCX*, thereby altering the balance of gene expression between *NRC2a/b* and *NRCX*.”

12. Page 14. “To our knowledge, this finding is a unique example where loss-of-function of….” As the authors did not investigate complete loss of function of NRCX, the authors should reconsider this conclusion. The authors only showed results of NRCX VIGS, raising a slight concern that phenotypes caused by NRCX VIGS and NRCX knockout might not be the same.

We toned down this sentence:

“To our knowledge, this finding is a unique example where VIGS of an *NLR* gene causes a “lethal” plant phenotype, following the definition by Lloyd et al. [47]. Therefore, *NRCX* is exceptional given that NLR genes tend to act just the opposite way, by causing fitness penalties.”

13. Page 14. “NRC2 and NRC3 partially suppress the dwarf phenotype….” Mutations in NRC2 and NRC3 do.

Thank you for pointing this out. We corrected this sentence as suggested.

14. Page 14. “This indicates that NRCX-mediated dwarfism is trigger-dependent……… other NLR(s) that ultimately perturbs plant growth.” The authors may explain this in more detail as readers might wonder what “the trigger” is. Can it be the virus used in VIGS? Or some environmental microbes? I do not understand “similar to pathogen recognition”.

We rephrased the sentence:

“This indicates that NRCX-mediated dwarfism may be dependent on existence of environmental microbes or viruses and may occur in particular tissues and/or during certain developmental stages.”

Reviewer #2:

This manuscript by Adachi et al. describes their functional analysis of an atypical Solanaceous helper NRC NRCX. In contrast to other NRCs, NRCX lacks a functional N-terminal MADA motif and the ability to trigger HR. Interestingly, silencing of NRCX in Nicotiana benthamiana lead to a dwarf phenotype, which is partially dependent on NRC2/3 but not NRC4. Moreover, RNA interference of NRCX enhances while overexpression of NRCX compromises NRC2 and NRC3-dependent cell death in N. benthamiana. These data suggest a negative role of NRCX in NRC2/3-mediated immune pathways/networks. Although the genetic data are largely convincing, biochemical supports are lacking. Here are some concerns the authors should address during revision.

Thank you very much for your evaluation. In this study, we mainly focus on genetic analyses of *NRCX* and report genetic role in NRC network context, and elected to submit our work to a genetics-focused journal (PLOS Genetics). To understand mechanism of NRCX-mediated modulation of the NRC network, we need to overcome technical difficulties of use of NRCX protein in biochemical and cell biology experiments. For instance, we so far faced low protein accumulation issue with NRCX. Such technical issue particularly affects biochemical and cell biology experiments. As a follow-up paper, we will improve this issue and will address mechanistic understanding of how NRCX modulates NRC2 and NRC3 subnetwork.

1. Fig 1. What is the evidence supporting that silencing of NRCX specifically causes the dwarf phenotype? Due to the various problems associated with vigs, the most reliable approach for corroboration is to generate knockout alleles of nrcx for phenotypic examination (the ko mutant is predicted to show the same or worse dwarfism than the vigs line). There is no description in the current manuscript on how many vigs lines were examined, and how reproducible the phenotype is. Does the vigs line affect the transcription or protein levels of NRC2/3 (Fig S4D is not quantitative enough to address mild transcriptional differences)? Answers to these questions may help explain how NRGX negatively regulate NRC2/3.

Thank you very much for your suggestion. Please refer to the response to the Editor above.

To show how reproducible the morphological phenotype of TRV:*NRCX* plants is, we added sentences describing the number of replicates to figure legends of Fig 1, Fig 5 and S6 Fig.

We performed RNA-seq analysis of TRV:*NRCX* samples, as shown in Fig 1C and 1D. In this analysis, *NRC2* and *NRC3* were not detected as differentially expressed genes in TRV:*NRCX* leaf, comparing to TRV:*GUS* control (S1 and S2 Files). Therefore, we speculate that the dwarf phenotype may not due to transcriptional up-regulation of helper *NRC* genes. We will address a question how NRCX modulates NRC2 and NRC3 subnetwork in future.

2. There is very limited analysis of the dwarf phenotype in the NRCX silencing line. Is it developmental or due to constitutive defense activation? What are its immune phenotypes (tested with pathogens)?

Thank you very much for your suggestion. To examine whether constitutive defense activation occurs in the *NRCX* silenced plants, we extracted total RNA from leaf tissue of TRV:*NRCX* and TRV:*GUS* plants and subjected to RNA-seq analysis. Comparing to TRV:*GUS* control, in total 3162 genes were differentially expressed in TRV:*NRCX* plants. Out of 3162 genes, 1445 genes were up-regulated and 1717 genes were down-regulated. Gene Ontology (GO) analysis revealed that GO terms ‘response to wounding’ and ‘response to biotic stimulus’ are significantly enriched in up-regulated genes. Therefore, we conclude that constitutive defense response is induced in the *NRCX* silenced plants. We have this data in Fig 1C and 1D and discussed in result:

“To determine whether constitutive defense activation occurs in the dwarf plants of TRV:*NRCX*, total RNA was extracted from leaf tissue of four-week-old TRV:*NRCX* and TRV:*GUS* plants and were subjected to RNA-seq analysis. In total, 3162 genes were detected as differentially expressed genes (DEGs, TRV:*NRCX* vs TRV:*GUS*), in which 1445 and 1717 genes were up-regulated and down-regulated, respectively (Fig 1C, S1 and S2 Files). The DEGs do not include NRC-H genes such as *NRC2*, *NRC3* and *NRC4* (S1 and S2 Files). Gene Ontology (GO) analysis showed significant enrichment of GO terms ‘response to wounding’ and ‘response to biotic stimulus’ in up-regulated DEGs, while ‘cell redox homeostasis’ is enriched in down-regulated DEGs (Fig 1D). DEGs having the GO terms ‘response to wounding’ and ‘response to biotic stimulus’ include genes encoding Proteinase inhibitor I and pathogenesis related proteins (S3 File). These results suggest that constitutive defense response is induced by *NRCX* systemic silencing, thereby resulting in the dwarf phenotype in *N. benthamiana* plants.”

3. Related to #2, how about the immunity related phenotypic analysis of the NRCX overexpression lines? Are they like the nrc2/3 knockout lines?

Thank you very much for your suggestion. As documented above, we performed RNA-seq analysis using TRV:*NRCX* and TRV:*GUS* plants and revealed that defense related genes are up-regulated in *NRCX* silenced plants. We think that the RNA-seq analysis is enough to examine defense related phenotype with *NRCX*.

3. From data in Fig 2-3, it seems that NRCX probably forms a non-functional N-terminal α1 helix and this may affect the activities of autoactive NRC2/3. It is possible that NRCX may serve as a dominant-negative form of helper NRCs, reminiscent of NRG1C reported recently. Thus, some simple protein-protein interaction experiments should be conducted to examine the interactions between NRCX and NRC2/3/4. The authors can test if NRCX competes with NRC2/3 to oligomerize, which may result in the formation of non-functional NRC resistosomes.

Thank you very much for constructive comments on understanding how NRCX modulates the NRC2/3 network. As we documented above, we need to overcome technical difficulties of use of NRCX protein in biochemical and cell biology experiments. We will improve this issue and will address the mechanistic insight as a future work.

4. In the abstract, the authors claim that NRCX has evolved to contribute to the homeostasis of the genetically unlinked NLR network. However, how it evolved is not clearly described in the manuscript. Is it co-existing with NRC2/3, and to what extent? From the phylogenetic tree, it seems that NRCX forms a small clade with a tomato NRCX homolog. To corroborate their conclusions on NRCX, the function of SlNRCX should be examined to see if it also works as a negative regulator.

Thank you very much for your suggestion. We now provide information about co-occurrence of NRCX with NRC2/3. We searched *NRCX* ortholog from other Solanaceae species (*Capsicum annuum*, *Capsicum chinense*, *Capsicum baccatum*, *Solanum commersonii*, *Solanum tuberosum*, *Solanum pennellii* and *Solanum chilense*) and closely related Convolvulaceae species (*Ipomoea triloba*, *Ipomoea nil* and *Ipomoea trifida*). In total, we identified 5 *NRCX* orthologs from 5 species (*N. benthamiana*, *S. commersonii*, *S. lycopersicum*, *S. pennellii* and *S. chilense*), although we failed to find *NRCX* ortholog from four Solanaceae reference genome databases (*C. annuum*, *C. chinense*, *C. baccatum* and *S. tuberosum*) (S5 Fig). Lack of *NRCX* ortholog gene in reference genome might be due to annotation issue. For instance, *NRC3* ortholog gene is annotated in previous version of tomato reference genome (ITAG 3.10) but not in the latest version (ITAG 4.0). We documented this result in “NRCX is a MADA-CC-NLR in the NRC helper phylogenetic clade” section. Moreover, we toned down our conclusion over the manuscript by removing the text that *NRCX* is an essential gene.

To examine the function of SlNRCX, we overexpressed wild-type SlNRCX with NRC3 autoactive mutant in *N. benthamiana* leaves. Overexpression of SlNRCX compromised cell death response by autoactive NRC3, in consistent with the function of NbNRCX (S12 Fig). We documented our new data in result:

“To further test whether tomato NRCX ortholog has a similar negative role in NRC3 autoactive cell death, we cloned wild-type *SlNRCX* and overexpressed it with NRC3^DV^ mutant in *N. benthamiana* leaves. Overexpression of wild-type SlNRCX compromised autoactive cell death response by NRC3^DV^, comparing to the empty vector control (S12 Fig). This result suggests that the modulator function of NRCX in NRC2/NRC3 networks is conserved between *N. benthamiana* and tomato.”

Minor points:

1. Page 3, explain what is CCR-NLRs before using the abbreviation.

We added the explanation.

2. Page5, the authors omitted an ‘it’ in the last paragraph. It should be ‘despite its sequence conservation, it probably forms a non-functional N-terminal α1 helix’.

We corrected this.

3. Page 8, change ‘HR and DV’ to ‘H474R and D475V’.

We corrected this.

4. Change ‘Nicotiana benthamiana’ to ‘N. benthamiana’ in the titles of each section.

We corrected this.

5. Page 12, to avoid confusion, the authors should use the bacterial strain name Pseudomonas fluorescens 55, which is virulent on N. benthamiana.

Thank you for pointing this out. We added the strain name.

Reviewer #3:

In their report, Adachi and coworkers describe the identification of a new paralogous NRC helper NLR in Nicotiana benthamiana and show functional analysis supporting its role as an attenuator of NLR-mediated immunity in solanaceous plants. Their observations are presented in the context of hybrid necrosis, NLRs’ phylogeny and evolution, tuning of ETI in plants and nicely couple with authors’ earlier work on NLR networking and their functional redundancy. The paper is clear and well written.

Thank you very much for your evaluation.

A few comments for author/editor consideration to further improve this production:

1. There is no information provided on possible off-target effects of silencing constructs. How unique were the sequences selected to trigger silencing? Were they the same for VIGS and dsRNA constructs?

Thank you very much for your suggestion. We used the same sequence for both VIGS and hpRNA constructs and documented in result and S2 Fig:

“This VIGS construct was made by using 5’ 446-bp sequence of *NRCX* gene that does not hit off-target candidate genes in Solanaceae Genomics Network VIGS Tool search (https://vigs.solgenomics.net/) using Nicotiana benthamiana v0.4.4 and Nicotiana benthamiana v1.0.1 databases (S2 Fig).”

2. In the model presented in Fig 8 the authors place NRCX downstream of NRC2&3 activation but the fact that no necroses were present after in-spot NRCX silencing may imply that NRCX associates with other NRCs prior to their activation; in other words: transient, hpRNA-mediated silencing might have not affected the complexes existing in the cell and it was not enough time for NRC2&3 to accumulate to levels triggering defenses. Is it possible that NRCX acts in fact upstream of other NRCs or competing with them for interactions w/ NRC-Ss? The autoimmunity of NRCs may be their “own” properties but it may also be a result of “oversensitive” sensors activating them in the absence of pathogen.

In the proposed model figure, we suggest NRCX may function downstream of NRC2/3. The main reason is that NRCX modulated NRC2/3 autoactive cell death response where sensor NLRs are not activated by cognate effectors. To address your question, we need to reveal molecular mechanism how NRCX modulates NRC2 and NRC3 networks. We will address this question as a future work.

3. The term of “RNA interference” (RNAi) was originally introduced in animals and indeed is sometimes used in plants interchangeably with post-transcriptional gene silencing but because two different techniques of gene silencing have been used (VIGS involves RNA interference too) I would abstain from using this term here and use “hairpin RNA-mediated Postranscriptional Gene Silencing” or “hpRNA-PTGS” instead. To emphasize the difference from VIGS (that affects the whole plant) and hpRNA-PTGS, the authors may also consider using simply “in spot” or ATTA-triggered silencing – just a suggestion. Note, that RNAi was also used in the Discussion.

Thank you very much for your suggestion about terms of gene silencing. As suggested, we changed the term “RNAi” to “hairpin RNA-mediated gene silencing” over the manuscript.

4. Minor: a few more reports involving crop plants may be added to the discussion where NLR-bearing loci are responsible for incompatibility and/or hybrid necrosis, for example the work involving wheat from B. Keller’s lab.

Thank you very much for your suggestion. Because we did not experimentally validate incompatibility and hybrid necrosis in F1 generation in our *N. benthamiana* system, we would like to keep our original discussion without the crop plant context.

Reviewer #4:

Review of PGENETICS-D-21-01645 “An atypical NLR protein modulates the NRC immune receptor network” by Adachi et al.

In this manuscript the authors provide evidence for the existence of a atypical CC-NLR of the N. benthamiana NRC class that negatively regulates the NRC immune network. The topic is nicely introduced and the introduction provides the reader with sufficient information to follow the conclusions. The experiments are very well performed and of high quality. The figures are very good and present the relevant data to follow the authors conclusions. The discussion is putting the results into the context of the current knowledge of NLR networks and enables the reader to further think about the issue tackled in the current manuscript.

The authors use VIGS in N. benthamiana to show that NRCX silencing induces a dwarf autoimmune related phenotype that is partially dependent on the presence of wildtype NRC2 and 3. They further show that changing NRCX expression levels affects NRC2 and 3, but not NRC4, function in sensor NRC-triggered immunity. The authors further suggest by swap-experiments between NRCX and NRC4 and by mutational analysis of NRCX that NRCX may present an atypical NLR (NRC NLR) that lacks cell death activity per se and may have evolved to regulate the NRC network. I really like this work and the manuscript, but I do have some suggestions to improve the claims and the manuscript.

Overall, I do think it is a nice piece of work out of the very productive Kamoun lab and provides great new insights into how NLRs are regulated and how they execute immunity.

below are some of my concerns and suggestion.

Thank you very much for your evaluation and comments.

main issues/questions:

abstract: An NRCX ‘sequence’-ortholog is not found in all Solanaceae species, right? Thus, it would suggest that NRCX has not evolved as an essential NLR NLR (NRC) gene in all Solanaceae. I suggest to specify this already in the abstract and to be clearer about this – NRCX regulates the NRC network in N. benthamiana, there might be other NLRs, not sequence ortholog to NbNRCX, regulating this network or there may be other regulatory mechanisms. What is the authors opinion on this?

Thank you very much for your suggestion. We searched *NRCX* ortholog from other Solanaceae species (*Capsicum annuum*, *Capsicum chinense*, *Capsicum baccatum*, *Solanum commersonii*, *Solanum tuberosum*, *Solanum pennellii* and *Solanum chilense*) and closely related Convolvulaceae species (*Ipomoea triloba*, *Ipomoea nil* and *Ipomoea trifida*). In total, we identified 5 *NRCX* orthologs from 5 species (*N. benthamiana*, *S. commersonii*, *S. lycopersicum*, *S. pennellii* and *S. chilense*), although we failed to find *NRCX* ortholog from four Solanaceae reference genome databases (*C. annuum*, *C. chinense*, *C. baccatum* and *S. tuberosum*) (S5 Fig). Lack of *NRCX* ortholog gene in reference genome might be due to annotation issue. For instance, *NRC3* ortholog gene is annotated in previous version of tomato reference genome (ITAG 3.10) but not in the latest version (ITAG 4.0). We documented this result in “NRCX is a MADA-CC-NLR in the NRC helper phylogenetic clade” section. Considering the possibility that there might be other modulator NLRs in Solanaceae plant species, we clarify and specify our sentences in the abstract by adding “in *N. benthamiana*”.

The experiments showing that NRCX and its alpha1 helix are unable to induce HR, even if swapped onto NRC4 DV is convincing (Fig.3). However, the follow up experiment testing whether NRCX MHD mutants can cause HR is (Fig. 4), at least to me, not the most logical one. I would have guessed that NRCX DV is unable to cause HR, because its N-terminal alpha helix is not (cell death) functional. Have the authors tried to see if NRCX or NRCX DV when its alpha 1 helix is swapped with the alpha 1 helix of NRC4 is able to cause cell death? This would give insights whether NRCX would in general be able to function in cell death or whether other constraints are in work.

Thank you very much for your constructive comments. First, we revised our story line of NRCX MHD mutant and MADA motif swap experiments by changing the order. Second, we generated MADA chimera constructs in the NRCX^DV^ and NRCX^HR^ MHD mutant backgrounds by using NRC2, NRC4 and ZAR1 MADA sequences. Although the NRCX chimeras have functional MADA sequences derived from the other MADA-CC-NLRs, the NRCX chimeras did not cause visible autoactive cell death response when expressed in *N. benthamiana* leaves. Therefore, we conclude that in addition to the non-functional MADA region, there is other constrain(s) in NRCX protein, thereby losing autoactive cell death activity unlike other helper NRCs. We discussed our new data in result and discussion:

“We also performed the opposite motif swap to test whether functional MADA sequences confer gain of cell death activity to NRCX. To this end, we produced MADA chimera constructs in the NRCX^DV^ and NRCX^HR^ MHD motif mutant backgrounds (S7A Fig). Although the N-terminal MADA sequences from NRC2 and ZAR1 are functional in NRC4^DV^ (Fig 4) [24], MADA^NRC2^-NRCX^DV^, MADA^ZAR1^-NRCX^DV^, MADA^NRC2^-NRCX^HR^ and MADA^ZAR1^-NRCX^HR^ did not induce any visible cell death when expressed in *N. benthamiana* leaves (S7B and S7C Fig). We further tested NRCX^DV^ and NRCX^HR^ chimera constructs with NRC4 MADA sequence (S7A Fig). Neither MADA^NRC4^-NRCX^DV^ nor MADA^NRC4^-NRCX^HR^ caused visible cell death response in *N. benthamiana* leaves (S7B and S7C Fig). These results suggest that in addition to the non-functional MADA region, there is other constrain(s) in NRCX protein that led to the incapacity to cause autoactive cell death unlike other helper NRCs.”

“NRCX clusters within the NRC-H subclade, which is populated with proteins with a MADA-CC-NLR architecture (Fig 2). However, the N-terminal MADA motif of NRCX was not functional when swapped into NRC4 (Fig 4), compared to other similar swaps we previously tested [10,24,66]. We conclude that the MADA motif of NRCX has degenerated from the canonical sequence present in the typical NRC helper NLRs and lost the capacity to execute the hypersensitive cell death response. In fact, a polymorphism in the NRCX MADA motif region, Thr-4, is unique to this protein among NRC-H (Fig 2). Moreover, given that functional MADA sequences did not confer cell death activity to NRCX (S7 Fig), NRCX may have become inactive to trigger cell death response by other mutation(s). Therefore, loss of cell death executor activity in NRCX might represent another path in the evolution of networked NLRs besides sensor and helper NLRs, as in the NLR functional specialization “use-it-or-lose-it” model described by Adachi et al. [24] and Kamoun [67]. We propose that NRCX has also functionally specialized into an atypical NLR protein that operates as an NLR modulator.”

Would it be possible to complement the severe dwarf phenotype of the NRCX silenced plants with a construct that would allow expression of a silencing resistant NRCX version? If yes, this would also make it possible to test whether NRCX P-loop function is required for the regulation or one could even test how a ‘auto-activation’ (MHD mutant version mimics potentially ATP binding oligomerization) of NRCX affects this regulation. This approach could be also used in the RNAi experiments, right? I think this would at least add up to the potential mechanism of how NRCX regulates NRC2/3.

Thank you for your suggestion. To perform complementation assay using silencing resistant NRCX version, we first need to make stable transgenic *N. benthamiana* plants expression the NRCX variant gene and then conduct systemic gene silencing experiment of *NRCX*. Considering that the suggested complementation experiment will take a lot of time, we would like to address this question in future.

When we used NRCX MHD or p-loop mutants in co-expression assay with autoactive NRCs, we observed phenotypic variations and could not make robust conclusion from the experiment. Therefore, we will address the molecular mechanism how NRCX modulates NRC2 and NRC3 as a future work.

Is a NRCX knock out plant viable? If not, can such a plant be generated in a nrc2/3 mutant background?

Thank you very much for your suggestion. Please see above for response to Editor on this point.

Did the authors get RNAseq data for NRCX silenced plant tissue? Are the expression levels of NRC2, 3 or any other NRCs changed in such a plant?

Thank you very much for your suggestion. To examine whether constitutive defense activation occurs in the *NRCX* silenced plants, we extracted total RNA from leaf tissue of TRV:*NRCX* and TRV:*GUS* plants and subjected to RNA-seq analysis. Comparing to TRV:*GUS* control, in total 3162 genes were differentially expressed in TRV:*NRCX* plants. Out of 3162 genes, 1445 genes were up-regulated and 1717 genes were down-regulated. NRC-H genes such as *NRC2*, *NRC3* and *NRC4* were not detected in the DEGs. Instead, Gene Ontology (GO) analysis revealed that GO terms ‘response to wounding’ and ‘response to biotic stimulus’ are significantly enriched in up-regulated genes. Therefore, we conclude that constitutive defense response is induced in the *NRCX* silenced plants. We have this data in Fig 1C and 1D and discussed in result:

“To determine whether constitutive defense activation occurs in the dwarf plants of TRV:*NRCX*, total RNA was extracted from leaf tissue of four-week-old TRV:*NRCX* and TRV:*GUS* plants and were subjected to RNA-seq analysis. In total, 3162 genes were detected as differentially expressed genes (DEGs, TRV:*NRCX* vs TRV:*GUS*), in which 1445 and 1717 genes were up-regulated and down-regulated, respectively (Fig 1C, S1 and S2 Files). The DEGs do not include NRC-H genes such as *NRC2*, *NRC3* and *NRC4* (S1 and S2 Files). Gene Ontology (GO) analysis showed significant enrichment of GO terms ‘response to wounding’ and ‘response to biotic stimulus’ in up-regulated DEGs, while ‘cell redox homeostasis’ is enriched in down-regulated DEGs (Fig 1D). DEGs having the GO terms ‘response to wounding’ and ‘response to biotic stimulus’ include genes encoding Proteinase inhibitor I and pathogenesis related proteins (S3 File). These results suggest that constitutive defense response is induced by *NRCX* systemic silencing, thereby resulting in the dwarf phenotype in *N. benthamiana* plants.”

minor issues

abstract: please explain the NRC abbreviation as you did for NLR proteins.

We added the abbreviation.

page 2 introduction: The sentence listing Arabidopsis NLR mutants is not correct – at least in my understanding. Should it not say: “Mutations in other Arabidopsis NLRs, such as laz5, adr1 ...are genetic suppressors of autoimmunity or cell death phenotypes.”? Because the phenotype depends on the wildtype genes and thus these cannot be genetic suppressors!

We corrected this sentence.

Figure 2: Please indicate what the red asterisks mean close to the labels of the truncated NLRs.

Thank you for pointing this out. We added the explanation in the figure legend.

Figure 4. According to my comment above in the main issue section I would suggest to move figure 4 to the supplement.

We changed the order of figures and revised the story line accordingly.

Results section for figure 4: I would disagree that this results clearly indicate that NRCX does not have the capacity to induce cell death. The alpha 1 helix of NRCX is insufficient and thus most likely the whole protein does not induce cell death, but I suppose if the alpha 1 helix would be swapped with the one of NRC4 this chimeric protein would be able to induce cell death – at least this should be tested to make this claim.

We responded to this comment above.

last paragraph of results: The authors write that P. fluorescence is an avirulent pathogen on N. benthamiana and that it triggers PTI. However, according to the general terminology an avirulent pathogen is considered to be detected by the plants NLR system and trigger ETI. Do the authors meant a non-virulent (not or only weakly growing/infecting) pathogen? Please explain.

We specified the bacteria with strain ID.

discussion: I would suggest to tone down the claim that NRCX is an essential plant gene, since the authors show it only for N. benthamiana and not for any other NRCX containing species.

We toned down our sentences over the manuscript as suggested.

Have all data underlying the figures and results presented in the manuscript been provided?

Large-scale datasets should be made available via a public repository as described in the PLOS Genetics data availability policy, and numerical data that underlies graphs or summary statistics should be provided in spreadsheet form as supporting information.

Reviewer #1: No:

Reviewer #2: Yes

Reviewer #3: Yes

Reviewer #4: None

We provided numerical data and statistics in S13 File.

PLOS authors have the option to publish the peer review history of their article (what does this mean?). If published, this will include your full peer review and any attached files.

If you choose “no”, your identity will remain anonymous but your review may still be made public.

Do you want your identity to be public for this peer review? For information about this choice, including consent withdrawal, please see our Privacy Policy.

Reviewer #1: No

Reviewer #2: No

Reviewer #3: Yes: Tadeusz Wroblewski

Reviewer #4: No
